# Protecting Frontline Health Care Workers from COVID-19 with Hydroxychloroquine Pre-exposure Prophylaxis: A structured summary of a study protocol for a randomised placebo-controlled multisite trial in Toronto, Canada

**DOI:** 10.1186/s13063-020-04577-8

**Published:** 2020-07-14

**Authors:** Julie K. Wright, Darrell H. S. Tan, Sharon L. Walmsley, Jennifer Hulme, Erin O’Connor, Carolyn Snider, Ivy Cheng, Adrienne K. Chan, Bjug Borgundvaag, Shelley McLeod, Michael H. Gollob, Rosemarie J. Clarke, Linda Dresser, Fatima Haji, Tony Mazzulli, Samira Mubareka, Peter Jüni, Dominic Lee, George Tomlinson, Kevin C. Kain, Megan Landes

**Affiliations:** 1 Division of Infectious Diseases, Department of Medicine, University of Toronto, Toronto, Ontario Canada; 2 Eliot Phillipson Clinician Scientist Training Program, Department of Medicine, University of Toronto, Toronto, Ontario Canada; 3 Department of Laboratory Medicine and Pathobiology, University of Toronto, Toronto, Ontario Canada; 4 Division of Infectious Diseases, Department of Medicine, University Health Network/Sinai Health System, Toronto, Ontario Canada; 5 Sandra Rotman Laboratories, Sandra Rotman Centre for Global Health, UHN-Toronto General Hospital, Toronto, Ontario Canada; 6 Institute of Health Policy, Management and Evaluation, University of Toronto, Toronto, Ontario Canada; 7 Division of Infectious Diseases, Department of Medicine, St. Michael’s Hospital, Toronto, Ontario Canada; 8 MAP Centre for Urban Health Solutions, St Michaels Hospital, Toronto, Ontario Canada; 9 Li Ka Shing Knowledge Institute, St. Michael’s Hospital, Toronto, Ontario Canada; 10 Immunodeficiency Clinic, Toronto General Hospital, University Health Network, Toronto, Ontario Canada; 11 Divisions of Emergency Medicine, Department of Family and Community Medicine, University of Toronto, Toronto, Ontario Canada; 12 Emergency Department, University Health Network, Toronto, Ontario Canada; 13 Divisions of Emergency Medicine, Department of Medicine, University of Toronto, Toronto, Ontario Canada; 14 Emergency Department, St. Michael’s Hospital, Toronto, Ontario Canada; 15 Emergency Department, Sunnybrook Health Sciences Centre, Toronto, Ontario Canada; 16 Division of Infectious Diseases, Sunnybrook Health Sciences Centre, Toronto, Ontario Canada; 17 Schwartz/Reisman Emergency Medicine Institute, Sinai Health, Toronto, Ontario Canada; 18 Division of Cardiology, Department of Medicine, University Health Network, Toronto, Ontario Canada; 19 Leslie Dan Faculty of Pharmacy, University of Toronto, Toronto, Ontario Canada; 20 Antimicrobial Stewardship Program, Sinai Health System/University Health Network, Toronto, Ontario Canada; 21 Investigational Pharmacy Services, University Health Network, Toronto, Ontario Canada; 22 Department of Microbiology, University Health Network / Sinai Health System, Toronto, Ontario Canada; 23 Sunnybrook Research Institute, Sunnybrook Health Sciences Centre, Toronto, Ontario Canada; 24 Department of Medicine, University of Toronto, Toronto, Ontario Canada; 25 Applied Health Research Centre (AHRC), St. Michael’s Hospital, Toronto, Ontario Canada; 26 Department of Medicine, University Health Network, Toronto, Ontario Canada

**Keywords:** COVID-19, Randomised placebo controlled trial, protocol, hydroxychloroquine, viral PCR, serology, psychological distress

## Abstract

**Objectives:**

*Primary Objective*: To determine if pre-exposure prophylaxis (PrEP) with 400mg hydroxychloroquine (HCQ), taken orally once daily reduces microbiologically confirmed COVID-19 among front line health care workers at high risk for SARS-CoV-2 exposure.

*Secondary Objectives*: To compare the following between study arms: adverse events; symptomatic COVID-19; duration of symptomatic COVID-19; days hospitalized attributed to COVID-19; respiratory failure attributable to COVID-19 requiring i) non-invasive ventilation or ii) intubation/mechanical ventilation; mortality attributed to COVID-19, number of days unable to work attributed to COVID-19, seroconversion (COVID-19 negative to COVID-19 positive over the study period); ability of participant plasma to neutralize SARS-CoV-2 virus *in vitro;*

To describe short-term psychological distress associated with risk of COVID-19 exposure at 1, 60, 120 days of the study.

To explore laboratory markers within participants with confirmed COVID-19: including circulating markers of host immune and endothelial activation in participant plasma and their correlation with disease severity and outcome

**Trial design:**

The HEROS study is a two-arm, parallel-group, individually randomized (1:1 allocation ratio), placebo controlled, participant and investigator-blinded, multi-site superiority trial of oral HCQ 400 mg taken once daily for 90 days as PrEP to prevent COVID-19 in health care workers at high risk of SARS-CoV-2 exposure. At 90 days, there is an open label extension wherein all participants are offered a one-month course of HCQ 400mg once daily for PrEP of COVID-19.

**Participants:**

Frontline HCWs aged 18 years of age or older, at high risk of SARS-CoV-2 exposure (including staff of emergency departments, intensive care units, intubation teams, COVID-wards, and staff deployed to Long Term Care facilities) of five academic hospitals in downtown Toronto, Canada.

Exclusion criteria include: currently pregnant, planning to become pregnant during the study period, and/or breast feeding; known hypersensitivity/allergy to hydroxychloroquine or to 4-aminoquinoline compounds; current use of hydroxychloroquine; known prolonged QT syndrome and/or baseline resting ECG with QTc>450 ms and/or concomitant medications which simultaneously may prolong the QTc that cannot be temporarily suspended/replaced; known pre-existing retinopathy, G6PD deficiency, porphyria, liver disease including cirrhosis, encephalopathy, hepatitis or alcoholism, diabetes on oral hypoglycemics or insulin, or renal insufficiency/failure; disclosure of self-administered use of hydroxychloroquine or chloroquine within 12 weeks prior to study; confirmed symptomatic COVID-19 at time of enrollment.

**Intervention and comparator:**

Intervention: hydroxychloroquine, 400mg (2 tablets) orally per day. Comparator: placebo, two tablets visually identical to the intervention, orally per day

**Main outcomes:**

The primary outcome is microbiologically confirmed COVID-19 (i.e. SARS-CoV-2 infection). This is a composite endpoint which includes positive results from any validated SARS-CoV-2 diagnostic assay including detection of viral RNA, and/or seroconversion.

Participants will be assessed at baseline, and then undergo monthly follow-up at day 30, 60, and 90, 120. At each visit, participants will provide an oropharyngeal sample, blood sample, and will undergo electrocardiogram monitoring of the QTc interval.

Secondary outcome measures include: adverse events; symptom duration of COVID-19; days of hospitalization attributed to COVID-19; respiratory failure requiring ventilator support attributed to COVID-19; mortality attributed to COVID-19; total days off work attributed to COVID-19; seropositivity (reactive serology by day 120); and short term psychological impact of exposure to SARS-CoV-2 at day 1, 60, 120 days using the K10, a validated measure of non-specific psychological distress.

**Randomisation:**

Within each site, participants will be individually randomized to either the intervention arm with HCQ or the placebo arm using a fixed 1:1 allocation ratio using an interactive web-based response system to ensure concealment of allocation. Randomization schedules will be computer-generated and blocked using variable block sizes.

**Blinding (masking):**

All participants, research coordinators, technicians, clinicians and investigators will be blinded to the participant allocation group.

**Numbers to be randomised (sample size)** N=988, randomised into two groups of 494 patients.

**Trial Status:**

**This summary describes protocol version No. 1.6, May 15, 2020.** Recruitment is ongoing - started April 20, 2020 and anticipated end date is July 30, 2021

**Trial registration:**

ISRCTN.com Identifier: ISRCTN14326006, registered April 14, 2020.

**Full protocol:**

The full protocol is attached as an additional file, accessible from the Trials website (Additional file 1). In the interest in expediting dissemination of this material, the familiar formatting has been eliminated; this Letter serves as a summary of the key elements of the full protocol.

The study protocol has been reported in accordance with the Standard Protocol Items: Recommendations for Clinical Interventional Trials (SPIRIT) guidelines (Additional file 2).

## Supplementary information

**Additional file 1.**

**Additional file 2.**

## Data Availability

Data will be made available from the author on reasonable request. Please contact Dr. Megan Landes (corresponding author).

