# Supplementary material for: Protecting Frontline Health Care Workers from COVID-19 with Hydroxychloroquine Pre-exposure Prophylaxis: A structured summary of a study protocol for a randomised placebo-controlled multisite trial in Toronto, Canada
Source: Trials. 2020 Jul 14;21:647. doi: 10.1186/s13063-020-04577-8 (PMC7359423; doi:10.1186/s13063-020-04577-8)
Supplement: Supplementary file 1 — Additional file 1. [file 13063_2020_4577_MOESM1_ESM.pdf]

**FULL TITLE:**	Protecting Frontline Health Care Workers from COVID-19 with Hydroxychloroquine Pre-exposure Prophylaxis: A Randomized, Placebo-controlled Multi-Site Trial in Toronto, Canada
**Protocol No.**	HEROS-1
**Principal-Investigator:**	Dr. Megan Landes
**Co-Principal Investigator**	Dr. Kevin Kain
**Study Support/Funding:**	National Research Council Canada<br>UHN Foundation<br>Apotex
**ISRCTN.com Identifier:**	14326006
**Version No.**	1.6
**Date:**	May 15, 2020

GCP Statement

This clinical study will be conducted in accordance with applicable Health Canada regulations, International Conference on Harmonisation of Technical Requirements for Registration of Pharmaceuticals for Human Use (ICH) guidelines on current Good Clinical Practice (GCP), and the Declaration of Helsinki.

Confidentiality Statement

This clinical study protocol contains information which is of a confidential, trade-secret or proprietary nature. The protocol is for the use of Sponsor-Investigators and their designated representatives participating in the investigational trial. It is not to be disclosed to any other person or party without the prior written approval of Sponsor-Investigator.

Table of Contents

INVESTIGATOR AGREEMENT **5**

STUDY CONTACT DETAILS **6**

ABBREVIATIONS AND DEFINITIONS **8**

PROTOCOL SYNOPSIS **11**

1.0 INTRODUCTION, BACKGROUND, AND STUDY RATIONALE **15**

1.1 Background 15
1.2 Current Treatment Options 15
1.3 Potential Risks and Benefits to Human Participants 16
1.4 Study Rationale 16

2.0 STUDY OBJECTIVES AND DESIGN **17**

2.1 Overall Study Design 17
2.2 Primary Objective 18
2.3 Secondary Objective(s) 18
2.4 Outcome measures 19

3.0 SELECTION AND ENROLLMENT OF PARTICIPANTS **20**

3.1 Number of Participants 20
3.2 Inclusion Criteria 20
3.3 Exclusion Criteria 21
3.4 Strategies for Recruitment 22
3.5 Enrollment Procedures 22
3.6 Co-enrollment Guidelines 22

4.0 WITHDRAWAL OF PARTICIPANTS **22**

4.1 Withdrawal criteria 22

5.0 RANDOMIZATION AND BLINDING PROCEDURES **23**

5.1 Randomization 23
5.2 Blinding 23

6.0 STUDY TREATMENTS **24**

6.1 Investigational Product 24
6.1.1 Description *24*
6.1.2 Expected Side Effects: *24*
6.2 Formulation and packaging 25
6.3 Placebo 25
6.3.1 Expected Side Effects *25*
6.3.2 Formulation and packaging *25*
6.4 Study Product Supply and Accountability 25
6.5 Dosing and Administration 26
6.6 Concomitant Medications 26
6.7 Dispensing Investigational Product to participants 26
6.7.1 Initial Visit *26*
6.7.2 Subsequent Study Visit *27*
6.7.3 Dispensing through non-UHN sites *27*
6.8 Participant Access to Study Medication at Study Closure 27

7.0 RISK MANAGEMENT **27**

7.1 Pregnancy and breastfeeding 27
7.2 Protection of Study Personnel from COVID-19 28
7.3 Drug Safety and Monitoring Board 28
7.4 Informing Participants of Amendments After Enrollment 29

8.0 CLINICAL AND LABORATORY EVALUATIONS 29

8.1 Clinical Evaluations 29
8.2 Laboratory Evaluations and Specimen Collection 30
8.2.1 Overview of Biologic Sample Collection 30
8.2.2 Microbiologic testing for SARS-CoV-2 31
8.2.3 SARS-CoV-2 Serology 31
8.2.4 Plaque reduction assay 31
8.2.5 Biomarkers of disease severity 31
8.3 Stored Research Specimens and Plans for Possible Future Testing 32
8.4 Questionnaires and/or Scales 32

9.0 STUDY PROCEDURES 34

9.1 Schedule of Events 34
Table 1: Schedule of Events 34
Special Schedule Considerations: 36
9.2 Information and Eligibility Session: (Day 0) - Conducted remotely 36
9.3 Baseline Visit: (Day 1) Conducted in-person 37
9.5 Weekly virtual questionnaire (Day 7 to day 120): Conducted remotely 38
9.6 Every Two-Weekly Viral Detection Sample Collection 39
9.7 Early Termination Visit 39
9.8 Re-contact of Participants after Trial Termination 39

10.0 EVALUATION AND REPORTING OF ADVERSE EVENTS 39

10.1 Adverse Event Reporting 39

11.0 STATISTICAL CONSIDERATIONS 41

11.1 General Study Design 41
11.2 Sample Size Considerations/Justification 41
11.3 Endpoints/Outcome Measures 41
11.3.1 Analysis of Primary Outcome Measures 41
11.3.2 Analysis of Secondary Outcome Measures 42
11.3.3 Interim Analysis 43

12.0 STUDY ETHICAL CONSIDERATIONS 44

12.1 Ethical Conduct of the Study 44
12.2 Informed Consent 44
12.3 Confidentiality 44
12.4 Research Ethics Board 45

13.0 GENERAL TRIAL CONDUCT CONSIDERATIONS 45

13.1 Adherence to Protocol 45
13.1.1 Protocol Amendments 45
13.1.2 Protocol Deviations 45
13.2 Monitoring & Auditing 46
13.2.1 Study Monitoring 46
13.3 Record Keeping 46
13.3.1 Data Collection 47
13.3.2 Data Corrections 47

13.3.3 Source Documents ... *47*
13.3.4 Data Management ... *47*
13.3.5 Record Retention ... *47*
13.4 Steering Committee ... 48

14.0 DISCLOSURE AND PUBLICATION POLICY ... 48

14.1 Publication of Study Results ... 48
14.2 Data Sharing for Secondary Research ... 48
14.3 Conflict of interest ... 48

15.0 REFERENCES ... 50

16.0 APPENDICES ... 54

16.1 Appendix 1: Patient Consent Document ... 54

INVESTIGATOR AGREEMENT

**Protocol Title:**	Protecting Frontline Health Care Workers from COVID-19 with Hydroxychloroquine Pre-exposure Prophylaxis: A Randomized, Placebo-controlled Multi-Site Trial in Toronto, Canada
**Protocol No.:**	HEROS-1
**Version No.:**	V1.6
**Date:**	May 15, 2020

This clinical study will be conducted in accordance with applicable Health Canada regulations, ICH guidelines on current GCP, and the Declaration of Helsinki.

I confirm that I have read and understand this protocol and I agree to conduct this clinical study in accordance with the design and specific provisions of the protocol, with the exception of a change intended to eliminate an immediate hazard to participants. Any deviation from the study protocol will be documented in the case report form.

I agree to promptly report to the applicable ethics boards any changes in the research activity and all unanticipated problems involving risks to human participants or others. Additionally, I will not make any changes in the research without prior ethics and sponsor approval, except where necessary to ensure the safety of study participants.

Name	Signature	Date (dd-mmm-yyyy)

STUDY CONTACT DETAILS

Role	Contact Details
Principal Investigator, Sponsor-Investigator	Megan Landes MD, MSc, CCFP-EM<br>Attending Staff, Emergency Department, University Health Network<br>Associate Professor, Dept. of Family and Community Medicine, University of Toronto<br>University Health Network, Toronto General Hospital<br>200 Elizabeth Street<br>R. Fraser Elliott Building, Ground Floor, Room 480<br>Toronto, ON M5G 2C4<br>Tel: 416-569-3429<br>Megan.landes@utoronto.ca
Co-Principal Investigator, Sponsor-Investigator	Kevin Kain MD, FRCPC<br>Director, SAR Laboratories, Sandra Rotman Centre for Global Health, and Director, Tropical Disease Unit<br>University Health Network, Toronto General Hospital<br>Professor, Department of Medicine, University of Toronto<br>MaRS, Toronto Medical Discovery Tower<br>101 College St, Ste 10-360A<br>Toronto, ON M5G 1L7<br>Tel: 416-581-7704<br>Kevin.kain@uhn.ca
Medical Monitor	Mona Loutfy MD, FRCPC, MPH<br>Professor, Infectious Diseases Specialist & Clinical Researcher, Women's College Research Institute, Women's College Hospital, University of Toronto & Maple Leaf Medical Clinic<br>Tel: 416-725-9566<br>Mona.Loutfy@wchospital.ca
SAE Reporting<br>Project Manager	Rosemarie J. Clarke RN, MHM, BScN, CHE, CCRP<br>Clinical Research Nurse Manager & Project Manager<br>University Health Network, Toronto General Hospital<br>585 University Avenue, 13 North,<br>Toronto, On, M5G 2N2<br>Tel: 416-340-4800 ext. 6723<br>Fax: 416-340-9105<br>Rosemarie.clarke@uhn.ca
Clinical Laboratory Facility	Kevin Kain MD, FRCPC<br>MaRS, Toronto Medical Discovery Tower<br>101 College St, Ste 10-360A<br>Toronto, ON M5G 1L7<br>Tel: 416-581-7704<br>Kevin.kain@uhn.ca

	T. Mazzulli, MD, FRCPC, FACP<br>Microbiologist-in-Chief<br>Department of Microbiology, Room 1487<br>Mount Sinai Hospital<br>600 University Ave.<br>Toronto, ON M5G 1X5<br>Tel. 416-586-4695<br>Fax. 416-586-8746<br>Email: tony.mazzulli@sinaihealthsystem.ca<br><br>Samira Mubareka, MD FRCPC<br>Sunnybrook Health Sciences Centre<br>2075 Bayview Ave., Room B1 03<br>Toronto, ON M4N 3M5<br>Tel: 416-480-4823, Fax: 416-480-6990<br>Samira.mubareka@sunnybrook.ca
Study Pharmacists	Linda Dresser Pharm D, FCSHP<br>Pharmacotherapy Specialist<br>Antimicrobial Stewardship Program<br>Sinai Health System/University Health Network<br>Tel: 416-488-0398<br>Linda.Dresser@uhn.ca<br><br>Fatima Haji BSc Pharm<br>Investigational Pharmacy Services<br>University Health Network<br>Tel: 416-340-3131 Ext 4562<br>Fatima.haji@uhn.ca
Data Coordination Centre	Applied Health Research Centre (AHRC)<br>Dominic Lee, Research Quality Lead<br>Tel: 416-360-4000 x77058<br>Dominic.Lee@unityhealth.to

ABBREVIATIONS AND DEFINITIONS

(Provide a list of abbreviations and definitions of unusual or specialized terms or measurement units used in the protocol. At the first appearance in the text – excluding summary - spell out abbreviated terms with the abbreviation indicated in parentheses.)

Acronym / Abbreviation	Definition
5-HT	5-hydroxytryptamine
ACE - 1, - 2	Angiotensin converting enzyme 1, 2
AE	Adverse Event
AGMP	Aerosol Generating Medical Procedure
AHRC	Applied Health Research Centre
ARI	Acute Respiratory Illness
C5a	Complement component 5a
CAD	Coronary Artery Disease
CHI3L1	Chitinase-3-like protein 1
COVID-19	Disease caused by the 2019 Novel Coronavirus, also known as 2019-nCoV or SARS-CoV-2
CQ	Chloroquine
CRF	Case Report Form
CRP	C-reactive protein
DAIDS	Division of AIDS
DM	Diabetes Mellitus
DSMC	Data Safety Monitoring Committee
eCRF	Electronic case report form
ELISA	Enzyme-Linked Immunosorbent Assay. This is a rapid immunochemical test that involves an enzyme and antibody or antigen
G6PD	Glucose-6-phosphate dehydrogenase
ICU	Intensive Care Unit
I-fabp	Intestinal fatty acid binding protein

IL-18, IL-18BP	Interleukin 18, Interleukin 18 binding protein
IL-6	Interleukin 6
IP	Investigational Product
HCQ	Hydroxychloroquine. Derivative of chloroquine, originally an anti-malarial medication
HCWs	Health care workers
HTN	Hypertension
LBP	Lipopolysaccharide-binding protein
LTC	Long term care
MD	Doctor of Medicine
MOH	Ministry of Health
MT	Mid-turbinate
NP	Nasopharyngeal (swab)
NP<br>*(as job title)*	Nurse Practitioner
PA	Physician Assistant
PEP	Post-exposure prophylaxis
PrEP	Pre-exposure prophylaxis
QTC	Corrected QT Interval
QT Interval	a measurement made on an electrocardiogram used to assess some of the electrical properties of the heart
REDCap	Research Electronic Data Capture system. Specific database used to store study data
REB	Research Ethics Board
RN	Registered Nurse
RNA	Ribonucleic acid
RPN	Registered Practical Nurse
RT-PCR	Reverse transcriptase–polymerase chain reaction
SAEs	Serious Adverse Events
SARS-CoV-2	Severe Acute Respiratory Virus Coronavirus 2

SHSC	Sunnybrook Health Sciences Centre
SC	Steering Committee
SDV	Source data verification
sEng	Soluble Endoglin
SHS	Sinai Health System
sICAM	Soluble Intercellular Adhesion Molecule
SMH	St. Michael's Hospital
SOP	Standard Operating Procedure
sTNF,<br>sTNFR2	Soluble Tumour Necrosis Factor,<br>Soluble Tumour Necrosis Factor receptor 2
(sTIE)1, sTIE2	Soluble Tyrosine kinase with immunoglobulin-like and EGF-like domains 1, 2
TGH	Toronto General Hospital
TNFα	Tumour Necrosis Factor alpha
TWH	Toronto Western Hospital
UHN	University Health Network
VEGF	Vascular Endothelial Growth Factor
WHO	World Health Organization
WOCP	Women of Child Bearing Potential

PROTOCOL SYNOPSIS

Full/Scientific Title	Protecting Frontline Health Care Workers from COVID-19 with Hydroxychloroquine Pre-exposure Prophylaxis:<br>a Randomized, Placebo-controlled Multi-Site Trial in Toronto, Canada
Short/Public Title	**HE**alth care worker p**RO**phylaxi**S** against COVID-19: The HEROS Trial
Protocol and Version	HEROS-1, v1.6
Primary Registry,<br>Trial Identifying No., Status,<br>Date of Registration	Clinical Trials Ontario<br>CTO Project ID: 2132; Approved<br>8 April 2020
Board of Record	University Health Network<br>REB chair: Dr. Morris Sherman, Dr.Morris.Sherman@uhn.ca
Secondary Identifiers	ISRCTN.com Identifier: 14326006
Clinical Phase	III
Health Condition Studied	Pre-Exposure Prophylaxis for COVID-19
Study Duration	Enrollment period: April 20, 2020 to July 30, 2021
	Study period: April 7, 2020 to January 30, 2022
Primary Sponsor-Investigators	Dr Megan Landes and Dr Kevin Kain
Contact: Public Queries	Elayna Fremes<br>200 Elizabeth Street<br>R. Fraser Elliott Building, Ground Floor, Room 480<br>Toronto, ON M5G 2C4<br>Tel: 416-340-4800 ext 5620
Contact: Scientific Queries	Dr. Megan Landes MD MSc CCFP-EM<br>megan.landes@utoronto.ca<br>University Health Network/Toronto General Hospital<br>200 Elizabeth Street<br>R. Fraser Elliott Building, Ground Floor, Room 480<br>Toronto, ON M5G 2C4<br>Tel: 416-569-3429
Countries of Recruitment	Canada
Number of Centres	5 sites in Toronto, Canada
Date of First Enrolment	30 April, 2020
Recruitment Status	Recruiting
Study Type	Interventional
Study Design	Investigator and participant-blinded, two-arm, parallel group, randomized placebo controlled superiority trial
Primary Objective	To determine if pre-exposure prophylaxis (PrEP) with 400mg hydroxychloroquine (HCQ), taken orally once daily reduces microbiologically confirmed COVID-19 among health care workers with high risk of SARS-CoV-2 exposure.
Secondary Objectives	To compare the following between study arms:<br>• Adverse events<br>• Symptomatic COVID-19<br>• Duration of symptomatic COVID-19<br>• Days hospitalized attributed to COVID-19

	• Respiratory failure attributable to COVID-19 requiring i) non-invasive ventilation or ii) intubation/mechanical ventilation<br>• Mortality attributed to COVID-19<br>• Number of days unable to work attributed to COVID-19<br>• Seroconversion (COVID-19 negative to COVID-19 positive over the study period)<br>• Ability of participant plasma to neutralize SARS-CoV-2 virus (plaque reduction neutralization test) *in vitro*<br>To explore laboratory markers within participants with confirmed COVID-19:<br>• Circulating markers of host immune and endothelial activation in participant plasma and their correlation with disease severity and outcome<br>To describe short-term psychological distress associated with risk of COVID-19 exposure at 1, 60, 120 days of the study.
Sample Size	N = 988
Allocation/ Randomization	Fixed 1:1 allocation ratio produced by a computer-based random number generator. Randomly permuted blocks of varying size will be used to ensure equal allocation to each group, stratified by site.
Study Population	Front line health care workers with high risk of SARS-CoV-2 exposure in Toronto, Canada
Key Inclusion Criteria	Health care worker (HCW) in clinical areas that pose high risk of exposure, anticipated to work at least 10 shifts over the study period (minimum 6 hours per shift) and anticipated to remain in their unit for the duration of the study;<br>age ≥18 years;<br>*For the purposes of the study:*<br>*"front line health care workers" are physicians (including residents), nurses, nurse practitioners, physician assistants, respiratory therapists, X-ray technicians, social workers and support staff (including but not limited to house-keeping, and porters).*<br>*"high risk of SARS-CoV-2 exposure" refers to units and/or teams expected to provide care to patients with COVID-19 and where the provision of this care is anticipated to require either aerosol generating medical procedures (AGMPs) or exposure to pre-symptomatic (i.e. unidentified) COVID-19 positive patients including (but not limited to) the Emergency Department, intubation response teams, Intensive Care Units, and response teams for Long Term Care (LTC) facilities and shelters served by participating hospital staff.*

Key Exclusion Criteria	1. Currently pregnant, planning to become pregnant during the study period, and/or breast feeding<br>2. Known hypersensitivity/allergy to hydroxychloroquine or to 4-aminoquinoline compounds.<br>3. Current use of hydroxychloroquine for the treatment of a medical condition.<br>4. Known prolonged QT syndrome and/or baseline resting ECG with QTc>450 ms (completed in the last 3 months) and/or concomitant medications which simultaneously may prolong the QTC that cannot be temporarily suspended/replaced.<br>5. Known pre-existing retinopathy, G6PD deficiency, porphyria, liver disease including cirrhosis, encephalopathy, hepatitis or alcoholism, diabetes on oral hypoglycemics or insulin, or renal insufficiency/failure.<br>6. Disclosure of self-administered use of hydroxychloroquine or chloroquine within 12 weeks prior to study.<br>7. Microbiologically confirmed symptomatic COVID-19 at time of enrollment
Investigational Product	Hydroxychloroquine (Apo-Hydroxyquine)
Control	Placebo
Administration and Dosing	400mg orally once a day
Duration of Treatment	Three months hydroxychloroquine vs placebo (Day 1-90) followed by one additional month open-label hydroxychloroquine for all participants (Day 90-120)
Outcome Measures	**Primary:** Microbiologically confirmed COVID-19 (i.e. SARS-CoV-2 infection). This is a composite endpoint which includes any validated SARS-CoV-2 diagnostic assay including detection of viral RNA, and seroconversion
	**Secondary Outcome Measures:**<br>a) Adverse events<br>b) Symptom duration of COVID-19<br>c) Days of hospitalization attributed to COVID-19<br>d) Respiratory failure requiring ventilator support attributed to COVID-19<br>e) Mortality attributed to COVID-19<br>f) Total days off work attributed to COVID-19<br>g) Seropositivity (reactive serology by day 120).<br>h) Short term psychological impact of exposure to COVID-19 at day 1, 60, 120 days.
Statistical Analysis	A Cox proportional hazards model, with a covariate for occupation (a proxy for individual risk of exposure) will be used to estimate the reduction in risk of the primary outcome associated with hydroxychloroquine. Follow-up time for this outcome will extend from randomization to infection or the end of the three-month randomized period (+ 14 days follow-up). One interim analysis will be carried out after half the expected number of infections (46/92) have occurred, with stopping for efficacy if the one-sided p-value for benefit is <0.0038 or futility if the one-sided p-value for benefit is > 0.51.
IPD Sharing Statement	Anonymized individual participant data collected during the trial, study protocols, statistical analysis plans and code, and informed consent documents, will be made available on reasonable request to researchers to achieve the ends of an approved, methodologically sound proposal. Proposals should be directed to the contact for scientific queries. To gain access, requestors will need to sign a

	data access agreement. Data will be available for 5 years following the close of the study.
Sources of Monetary/Material Support	The National Research Council of Canada, The Thistledown Foundation, The GeoSentinel Foundation, The Slaight Family Foundation<br>Apotex donated the study drug and placebo.

1.0 INTRODUCTION, BACKGROUND, AND STUDY RATIONALE

1.1 Background

On March 11th, 2020, the World Health Organization (WHO) declared coronavirus disease (COVID-19) caused by severe acute respiratory virus coronavirus 2 (SARS-CoV-2) a pandemic.[1] As of March 22, 2020 there have been over 267,013 confirmed cases and 11,201 deaths in 185 different countries or regions;[2] it is quickly overwhelming health care systems worldwide to detrimental effect. Even with current moderate interventions imposed, estimates predict that 10-18% of the Canadian population will be infected with the virus by its peak in July.[3] Health care workers (HCWs) remain a critical line of defence in the fight against this pandemic and maintaining their health is not only an important social responsibility of the government, but it is also of vital national interest to treat and control others infected with this virus or sick with other disease.

Estimates from China indicate that HCWs make up 3.8% of cases,[4] while in Italy it is reported to be much higher – 8.3%.[5] Early numbers from front-line workers in Italy suggested up to 20% of HCWs become infected with COVID-19,[6] and these rates are approximately 3 times higher than the general population.[7] When HCWs become sick, not only do they risk dying, but they also must take weeks away from work limiting the ability of the health care system to function. The fear associated with becoming ill also causes higher rates of missed work and higher rates of burnout, as seen with previous outbreaks.[8] It is also now clear that asymptomatic transmission of COVID-19 not only occurs,[9] but may even be the most important factor in spread of the virus.[10] HCWs may therefore become vectors of viral spread to those who are the most vulnerable in other areas of the hospital. Preventing our HCWs from acquiring SARS-CoV-2 should therefore be of the utmost importance to our national interests.

1.2 Current Treatment Options

Repurposing drugs already known to be safe and tolerable in humans provides a major advantage in a pandemic where time is critical. Of candidate drugs, chloroquine (CQ) and its derivative hydroxychloroquine (HCQ), have shown some promise. Originally an anti-malarial medication, CQ exerts direct antiviral effects by inhibiting pH-dependent steps of the replication of several viruses, including coronaviruses.[11] It also has immunomodulatory effects, suppressing the release of TNFα and IL-6, which are involved in the inflammatory complications of several viral diseases.[12] *In vitro* data has shown that CQ potently blocks virus infection at low micromolar concentrations with a high selectivity index.[13] CQ is also widely distributed throughout the body, including the lungs, after oral administration.[13] An early clinical trial of more than 100 COVID-19 patients in China reported that CQ was superior to placebo in inhibiting pneumonia, improving lung

imaging, promoting viral seroconversion, and shortening the disease course, although data was not released.[14] Based on those results experts in China recommended CQ 500mg twice daily for ten days in all patients with COVID-19.[15] HCQ has also been shown to also have anti-SARS-CoV-2 activity *in vitro* and may actually be more potent.[16] It has a better safety profile than CQ (during long term use), and allows a higher daily dose,[17] with fewer concerns of drug-drug interactions.[18] Preliminary clinical data also suggests that HCQ may lead to significantly faster viral clearance in COVID-19 patients as assessed by nasopharyngeal swab (70% vs. 12.5%, p=0.001).[19]

1.3 Potential Risks and Benefits to Human Participants

CQ and HCQ have a long history of safety when used for both malaria prophylaxis and in rheumatic disease. Very commonly occurring (>10%) adverse events include gastrointestinal (abdominal pain and nausea), with less commonly (1-10%) occurring events including blurring of vision, diarrhea, vomiting, anorexia, headache, emotional lability and skin rash**.** There is a low incidence of serious adverse events, most commonly occurring during chronic use (>5 years), with the main risk being macular retinopathy (<1%), which depends on the cumulative dose and permanent damage can be prevented with regular vision exams during treatment.[20-23] Other rare events (<1%) include liver dysfunction, hypoglycemia and while HCQ can increase the QT interval, the frequency of this event is not known, but can be increased when used in conjunction with other drugs that prolong the QT interval. It has shown safe even in higher doses (up to 500mg/day of CQ base).[24]

There may be no medical benefit to participants from participating in this trial as the efficacy of HCQ in the prevention of COVID-19 has yet to be shown. There may be non-medical benefits in contributing to research that might benefit others, and other emotional and social benefits, including empowerment that participants are taking another measure to potentially reduce their risk of infection of COVID-19.[25]

1.4 Study Rationale

Currently, there are studies underway looking at treatment of COVID-19 with HCQ, as well as post-exposure prophylaxis (PEP); to our knowledge there are no studies of HCQ, or any other antiviral, for pre-exposure prophylaxis (PrEP). PrEP offers the ability to protect front-line HCWs from illness, decrease nosocomial spread of SARS-CoV-2, and prevent loss of work force due to illness. Rigorous testing of HCQ for this purpose is critical at this time. We therefore aim to conduct the first randomized placebo-controlled trial of HCQ to prevent COVID-19 infections in front line health care workers at high risk of SARS-CoV-2 exposure as we prepare for escalating rates of COVID-19 in Toronto, Canada.

2.0 STUDY OBJECTIVES AND DESIGN

2.1 Overall Study Design

The HEROS study is a double-blind, randomized placebo controlled trial of oral HCQ 400 mg taken once daily for three months as PrEP to prevent COVID-19 in front line health care workers at high risk for SARS-CoV-2 exposure.

The trial will recruit 988 HCW at high risk of COVID-19 exposure from five academic hospitals in downtown Toronto: University Health Network (UHN) [Toronto General Hospital (TGH) / Toronto Western Hospital (TWH)], St. Michael's Hospital (SMH), Sinai Health System (SHS), and Sunnybrook Health Sciences Centre (SHSC).

Within each site, participants will be randomly assigned to either the intervention arm with HCQ or the placebo arm using a fixed 1:1 allocation ratio produced by a computer-based random number generator using variable block sizes to ensure equal allocation to each group.

The rationale for conducting a randomized placebo-controlled trial is:
first, little is known to date regarding the effectiveness of HCQ against SARS-CoV-2 *in vivo*, yet *in vitro* studies signal plausibility of virucidal activity;
second, no data exist supporting the efficacy of any biomedical interventions in health care workers for preventing COVID-19.

Participants will be seen at baseline, and then undergo monthly follow-up at day 30, 60, and 90, 120. The primary outcome will be symptomatic COVID-19 (with microbiological confirmation of SARS-COV-2 infection) anytime within 104 days (= the 90 days of the randomization period plus 14 additional days to allow for viral incubation, delayed symptom onset and/or seroconversion).

At 90 days an open label extension wherein all participants are offered a one-month course of HCQ 400mg once daily for PrEP of COVID-19. The rationale for the open-label extension is that it will improve participant adherence to their allocation group during the randomization period. It will further allow additional evidence regarding adherence and safety. A period of one month will be offered as this is the amount of drug that is available for the study.

A Cox proportional hazards model, using a covariate for occupation (a proxy for risk of exposure) will be used to estimate the reduction in risk of the primary outcome associated with hydroxychloroquine use. Follow-up time for this outcome will extend from randomization to the first of: infection, or the end of the three-month randomized period (+ 14 days). One interim analysis will be carried out after half the expected number of infections (46/92) have occurred, with stopping for efficacy if the one-sided p-value for benefit is <0.0038 or futility if the one-sided p-value for benefit is > 0.51. If efficacy is shown at this time, all participants will be offered open-label HCQ until the end of the full study period (Day 120). If the study is not stopped at the interim analysis and HCQ has not otherwise been shown effective at that time, all study participants will continue to receive blinded study drug until day 90, and then be offered one month of HCQ in an open-label extension (until day 120).

2.2 Primary Objective

To determine if pre-exposure prophylaxis (PrEP) with 400mg hydroxychloroquine (HCQ), taken orally once daily reduces microbiologically confirmed COVID-19 among front line health care workers at high risk for SARS-CoV-2 exposure.

2.3 Secondary Objective(s)

1. To compare the following secondary outcomes between study arms:

- Adverse events
- Duration of symptomatic COVID-19
- Days hospitalized attributed to COVID-19
- Respiratory failure attributable to COVID-19 requiring i) non-invasive ventilation or ii) intubation/mechanical ventilation
- Mortality attributed to COVID-19
- Number of days unable to work attributed to COVID-19
- Asymptomatic COVID-19
- Ability of participant plasma to neutralize SARS-CoV-2 virus (plaque reduction neutralization test) *in vitro*

2. To explore laboratory markers within participants with confirmed COVID-19:

- Severity markers of host immune and endothelial activation measured at clinical presentation and their ability to predict severity and outcome

3. To describe short-term psychological distress associated with risk of COVID-19 exposure at 1, 60, 120 days of the study.

2.4 Outcome measures

The **primary outcome is microbiologically confirmed COVID-19 (SARS-CoV-2 infection).** This is a composite endpoint which includes any positive result from a validated SARS-CoV-2 diagnostic assay including detection of viral RNA, or seroconversion by day 104 (14 days after end of the randomization period).

The microbiologic outcome measures will be obtained from validated diagnostic assays performed on participant samples (viral detection samples or blood) collected at defined intervals over the course of the study (see Section 9.1 and Table 1, Schedule of Events). Symptomatic outcome measures, clinical outcomes (ex. hospitalization, need for supported ventilation), days unable to work etc. will be collected by self-report *via* weekly email survey (see Section 9.1 Table 9.1, Schedule of Events). In the case of death or other condition rendering the participant unable to communicate or complete the weekly email survey, the outcome will be obtained by contacting the designate identified by the participant at enrollment. If it is not possible to reach this person, the participant will be considered lost to follow up.

Per Public Health Agency of Canada, Public Health Ontario, and hospital guidelines, during this COVID-19 pandemic, all HCWs must report to occupational health and their departmental supervisors and self-isolate (i.e. they must not come to work) should they develop any signs or symptoms of COVID-19, at which time they will be prioritized for testing either in the emergency department or at occupational health (and less frequently at MOH testing centres), as per site institutional guidelines. Diagnostic testing will be prompted by symptoms and be conducted according to these routine channels. Results of these tests will be collected by the data collection measures in this study.

Secondary outcomes measures are:

a) **Adverse events:** as defined using the DAIDS Table for Grading the Severity of Adverse Events, until day 120[27]

b) **Symptom duration of COVID-19:** measured in days.

c) **Days of hospitalization attributable to COVID-19:** The number of days (or partial days) spent admitted to an acute care hospital during the study period.

d) **Respiratory failure requiring ventilatory support attributable to COVID-19:** The number of days (or partial days) requiring i) non-invasive and ii) endotracheal intubation with ventilation during the study period.

e) **Mortality** attributable to COVID-19 and all-cause mortality during the study period.

f) **Number of days ineligible/unable to work due to COVID-19:** measured from symptom onset to return to work date.

g) **Seropositivity:** reactive serology by day 120.

h) **Short-term psychological impact of exposure to COVID-19** will be measured at the beginning of randomization (day 1), during the randomized period (day 60) and during the open-label period (day 120) using the K10, a validated measure of non-specific psychological distress, with a standard cutoff score of ≥16.[27,28]

3.0 SELECTION AND ENROLLMENT OF PARTICIPANTS

3.1 Number of Participants

The trial will enroll 988 participants across all five hospital sites in Toronto, Canada.

3.2 Inclusion Criteria

1. Health care worker (HCW) in clinical areas that pose high risk of exposure, who is anticipated to work at least 10 shifts over the duration of the study period (minimum 6 hours per shift) and anticipated to remain in their unit for the duration of the study (i.e., not transferring to another unit).

For the purposes of the study:

- *"front line health care workers" are physicians (including residents), nurses, nurse practitioners, physician assistants, respiratory therapists, X-ray technicians, social workers and support staff (including but not limited to house-keeping, and porters).*
- *"high risk of SARS-CoV-2 exposure" refers to units and/or teams expected to provide care to patients with COVID-19 and where the provision of this care is anticipated to require either aerosol generating medical procedures (AGMPs) or exposure to pre-symptomatic (i.e. unidentified) COVID-19*

positive patients including (but not limited to) the Emergency Department, intubation response teams, Intensive Care Units, and response teams for Long Term Care (LTC) facilities and shelters served by participating hospital staff.

2. Age ≥18 years.
3. Ability to communicate with study staff in English

3.3 Exclusion Criteria

8. Currently pregnant, planning to become pregnant during the study period, and/or breast feeding
9. Known hypersensitivity/allergy to hydroxychloroquine or to 4-aminoquinoline compounds.
10. Current use of hydroxychloroquine for the treatment of a medical condition.
11. Known prolonged QT syndrome and/or baseline resting ECG with QTc>450 ms (completed in the last 3 months) and/or concomitant medications which simultaneously may prolong the QTC that cannot be temporarily suspended/replaced. These are including but not limited to Class IA, IC and III antiarrhythmics; certain antidepressants, antipsychotics, and anti-infectives; domperidone; 5-hydroxytryptamine (5-HT)3 receptor antagonists; kinase inhibitors; histone deacetylase inhibitors beta-2 adrenoceptor agonists.
12. Known pre-existing retinopathy, G6PD deficiency, porphyria, liver disease including cirrhosis, encephalopathy, hepatitis or alcoholism, diabetes on oral hypoglycemics or insulin, or renal insufficiency/failure.
13. Disclosure of self-administered use of hydroxychloroquine or chloroquine within 12 weeks prior to study. This window allows five half-lives of HCQ (i.e. 21 days) to pass before being reintroduced to the drug.
14. Confirmed symptomatic COVID-19 at time of enrollment, i.e. symptom of COVID-19 at enrollment with confirmation of SARS-CoV-2 infection by viral detection as performed according to local guidelines for symptomatic HCWs. All participants with COVID-19 symptoms at enrollment will be directed to have confirmatory testing (within the department or occupational health as per the site guidelines). Participants who are negative for SARS-CoV-2 will be redirected to enrollment procedures; those testing positive will be excluded.

Site investigators are required to assess any concomitant medications being taken at the time of study enrollment to ensure they are not exclusionary based on criterion #3 above. In cases of uncertainty, the investigator is encouraged to consult with the study pharmacist (see contact information in section Study Contact Details, page 6).

3.4 Strategies for Recruitment

Eligible staff will be invited *via* email to an online information forum in which study information and eligibility will be discussed in general. In addition to this email, a poster outlining a brief overview of the study and contact information will be displayed in the hospital sites in areas and departments where relevant cadres of HCW will view the poster. This is congruent with currently recommended physical distancing practices. Staff willing to participate and who feel that they meet eligibility criteria will be able to schedule an individual appointment with study coordinators over subsequent days which will provide them with the opportunity to ask questions about participation, confirm eligibility, sign a written consent form, and complete an enrollment visit/procedures.

3.5 Enrollment Procedures

Study staff will complete enrollment procedures with participants in a private room. Enrolment will be scheduled on a first come, first served basis over the pre-specified scheduled days. Any participant screening positive for symptoms of COVID-19 (see Symptom Screen in Questionnaire Content/Appendix B) will be directed to the most appropriate place for a confirmatory COVID-19 test (within the emergency department or occupational health as per site institutional guidelines). Of these, participants who have a negative COVID-19 screen will be redirected to enrollment procedures, persons with a positive COVID-19 screen will be excluded.

The consent process will involve obtaining a signed copy of the REB-approved written informed consent form. Participants will be interviewed by study staff, asked to submit a baseline socio-demographic questionnaire, have a venous blood sample drawn, and provide a self-collected viral detection specimen.

3.6 Co-enrollment Guidelines

Co-enrollment into other investigational prevention studies will not be permitted at this time.

4.0 WITHDRAWAL OF PARTICIPANTS

4.1 Withdrawal criteria

Participants may be withdrawn from the study if the Sponsor-Investigator terminates the trial (i.e. if new evidence emerges proving either futility or efficacy of the study drug or

potential safety concerns or if drug supply becomes an issue). Grade 3 or 4 adverse events or an SAE may warrant withdrawal from the study. If a new health condition emerges in a participant randomized to the HCQ arm that requires medications absolutely contraindicated by the protocol, then decisions regarding discontinuation of the study drug can be made at the discretion of the site investigator, but the participant should be retained in the study for ascertainment of the primary and secondary outcomes. Site investigators are encouraged to consult with the study pharmacist in such situations. Reasons for the discontinuation of study drug will be noted in the study database. If the participant discloses that they have been self-administering any other medication for the prevention of COVID-19, it will be noted in the database and the participant will be retained in the study for ascertainment of primary and secondary outcomes. If a participant withdraws from the medication, with consent, the participant will still be followed for primary and secondary outcomes.

5.0 RANDOMIZATION AND BLINDING PROCEDURES

5.1 Randomization

Once a participant signs the informed consent document, the Research Coordinator will issue a Study ID number and enter the Study ID into a secure web-based randomization system that will be housed at the Applied Health Research Centre (AHRC) at St. Michael's Hospital. Randomization will occur through this central system which provides adequate allocation concealment to perform randomization. Randomization will be in a 1:1 ratio with permuted blocks of variable size and stratified by study site. The randomization sequences will be pre-determined by a non-trial statistician who will be the only person with access to the study codes. An automated audit trail will record the time, date, allocation, and participant identification numbers.

5.2 Blinding

This trial is fully-blinded, at the level of the participant, study coordinator, and investigator. Study drug and placebo will be identical in appearance and will be provided by the manufacturer. They will be packaged in identical bottles and dispensed by the Research Clinical Trials Pharmacy at UHN.

Blind will only be broken if an immediate safety issue arises for a participant in which knowledge of the treatment allocation will impact clinical care. The Medical Monitor of the study must be consulted in these situations.

When interim analyses are conducted, the study coordinator will send relevant data from participants enrolled and the codes to be included in the interim analysis to the biostatistician working with the Data Safety and Monitoring Board.

6.0 STUDY TREATMENTS

6.1 Investigational Product

6.1.1 Description

Brand or Product Name: Apo-Hydroxyquine
Manufacturer: Apotex
Generic Name: Hydroxychloroquine
DIN - 02246691

Hydroxychloroquine (HCQ) sulfate tablets belong to the 4-aminoquinoline class. HCQ has been beneficial for patients with rheumatoid arthritis and lupus erythematosus, especially chronic discoid lupus. The exact mode of action in controlling these diseases is unknown. The action of this compound against malarial parasites is similar to that of chloroquine phosphate: interferes with glycosylation of cellular ACE2 receptor (COVID-19 uses ACE2 receptors to bind to target cells), impairs acidification of endosomes which is required for virus/cell fusion, along with other immunomodulatory effects.

6.1.2 Expected Side Effects:

Most side effects are seen in treatment doses and in long term use (over years). Most are reversible with stopping the medication (unless otherwise indicated):

Very Common: (>10%): Abdominal pain, nausea

Common: (1-10%): Blurring of vision due to a disturbance of accommodation, diarrhea, vomiting, anorexia, headache, affect lability, skin rash/pruritis,

Uncommon: <1%: Vertigo, tinnitus, maculopathies (in early form is reversible), abnormal liver function tests, dizziness, nervousness, pigmentary changes in skin, hair

Not known (event rate): blood disorders, cardiomyopathy, prolonged QTC, urticaria, angioedema, bronchospasm, myopathy

Serious: Most serious toxicities are associated with long term use (ie. over years) and include: retinopathy, liver dysfunction, and hemolysis in G6PD deficient patients (NB: Product monograph suggests G6PD testing prior to initiation but post-marketing studies suggest risk is low and in standard clinical practice this is not done.)

6.2 Formulation and packaging

The manufacturer Apotex will provide study medication (hydroxychloroquine and matching placebo). The pharmacy department of the University Health Network will be responsible for labelling and distribution of study medication. Study medication will be stored by the pharmacy in accordance with relevant guidelines. The study medication is supplied in tablet formulation and dispensed in bottles labelled in the following way: dispensing label (including patient name, directions for use, prescriber) and the Health Canada label (to meet standards for investigational drug).

6.3 Placebo

Matched placebo will be provided from the manufacturer Apotex.

6.3.1 Expected Side Effects

There are no expected side effects from the placebo.

6.3.2 Formulation and packaging

Placebo will be provided in the same format as described above for the interventional drug.

6.4 Study Product Supply and Accountability

All study medications will be stored at 15-30°C in a secure location. Study medication will be collected from the pharmacy department by the principal investigator or his/her delegate. Unused or partially used investigational products (IP) will be returned to the study coordinator. Destruction of the IP via Stericycle will be coordinated with the site pharmacy of each participating institution

The study medication should be stored in a secure area according to local regulations. The investigator is responsible for ensuring that it is dispensed only to study subjects and only from official study sites by authorized personnel, as dictated by local regulations.

The investigator is responsible for ensuring that the study medication is stored under the appropriate environmental conditions (temperature). If concerns regarding the quailty or

appearance of the study medication arise the study product will not be dispensed and Apotex will be contacted immediately.

6.5 Dosing and Administration

During the randomization period (90 days), HCQ will be administered as 400mg orally once daily in the treatment arm and placebo tablets will be administered similarly once daily for 90 days. The open-label extension period will offer all participants HCQ 400 mg orally once daily for one month.

6.6 Concomitant Medications

Study investigators must review concomitant medications at enrollment as part of eligibility criteria for enrollment. Investigators are encouraged to consult with the study pharmacist in cases of uncertainty. All other concomitant medications will be collected at study visits and recorded.

6.7 Dispensing Investigational Product to participants

6.7.1 Initial Visit

Once a participant signs the informed consent document, the Research Coordinator will issue a Study ID number and enter the Study ID into a secure web-based randomization system (REDCap) that will be housed at the Applied Health Research Centre (AHRC) at St. Michael's Hospital. Randomization will occur through this central system which provides adequate allocation concealment to perform randomization. Randomization will be in a 1:1 ratio with permuted blocks of variable size and stratified by study site. The randomization sequences will be pre-determined by a non-trial statistician. The randomization code will be a 6 digit number not related to the participant's study ID number. An automated audit trail will record the time, date, allocation, and participant identification numbers.

Once the study coordinator has the participant's randomization code, this will be communicated to the clinical trials pharmacy by email. The clinical trials pharmacist will dispense the appropriate investigational product (IP) by cross referencing the randomization code of the participant to the randomization list provided by the trial statistician. The study coordinator will pick up the IP bottles from the clinical trials pharmacy at an agreed upon time and deliver it to the study participant.

6.7.2 Subsequent Study Visit

For subsequent study visits, the study coordinator will email the clinical trials pharmacy 24 hours prior to the scheduled visits to request the IP bottles required. The clinical trials pharmacy will dispense the requested IP bottles to the study coordinator who will dispense them to the participant at the study visit.

6.7.3 Dispensing through non-UHN sites

For additional trial sites, the central pharmacy at UHN will prepare the IP according the randomization codes and ship to the pharmacies of other sites via courier. Local Standard Operating Procedures for accountability will be verified and approved prior to issuing and dispensing IP through these sites.

6.8 Participant Access to Study Medication at Study Closure

At the end of the 90-day randomization period, all participants will be offered a 30-day open-label extension of hydroxychloroquine 400 mg orally daily. Beyond the study period, HCQ is not currently indicated for routine prophylaxis of viral infections. If the study is terminated for efficacy, all participants will be offered the 30-day open-label extension at that time.

In the event that a study participant tests positive for COVID-19, participants will be instructed to stop their study drug. If this occurs during the randomization period, neither participant nor study staff will not be unblinded to allocation group. Efforts will be made to link the individual to ongoing clinical trials of COVID-19 treatment if such trials are enrolling in proximity to the study site and the participant meets the inclusion criteria. However, no guarantees of access to investigational product can be made. At the time of writing, there are no known effective treatments for established COVID-19 infection.

7.0 RISK MANAGEMENT

7.1 Pregnancy and breastfeeding

Health Canada has not approved the use of HCQ for women who are currently pregnant, planning to get pregnant, or who are breastfeeding. Women meeting these criteria at the screening will be ineligible to enroll. Further, women of childbearing potential (WOCP) will have a screening urine pregnancy test and only those with negative tests will be enrolled. Urine pregnancy tests will be done on WOCP at each study visit. Women who become pregnant during the study will be asked to inform the study coordinator and to stop their study drug and will discuss follow-up with the participant. All WOCP at the

beginning of the study will agree to the use of a contraceptive method. Pregnancy is not an adverse event; however, any complication related to pregnancy should be considered an adverse event.

7.2 Protection of Study Personnel from COVID-19

COVID-19 is believed to be transmitted from person-to-person through respiratory droplets and via fomites. While the greatest risk of transmission is believed to occur in the context of respiratory symptoms, transmission from asymptomatic and minimally symptomatic individuals may occur. For these reasons, several measures will be taken to minimize the risk of transmission to study personnel and participants.

First, the group information session for consent will be conducted remotely. Weekly online questionnaires will collect the majority of data required from participants, further reducing person-to-person contact in the monthly interview. For study visits conducted in person, if the participant does not have symptoms of COVID-19 (see Questionnaire Content document), universal precautions will be undertaken for the interview and phlebotomy. If the participant fails the symptom screen, a surgical mask should be placed on the participant and full droplet precautions will be followed by study staff (i.e. surgical mask, eye protection, gown, glove). Any study staff person who develops symptoms potentially suggestive of COVID-19 should immediately be assessed by the site occupational health department and relieved of study duties until deemed safe to return to work.

7.3 Drug Safety and Monitoring Board

Risk minimization, management, and assessment procedures have been implemented in the study to minimize and assess potential risks to participants who participate in this clinical study with HCQ. Components include: (1) specific study entry and exclusion criteria to ensure that participants who have underlying condition that potentially increase their risk for an adverse outcome are excluded; (2) overview surveillance by an independent Data Safety Monitoring Board (DSMB); (3) follow-up (monthly) for safety monitoring purposes at in-person study visits.

The DSMB will include infectious diseases and public health specialists, at least one biostatistician, and researchers with an understanding of emerging infections and clinical trials, not involved in the trial. The DSMB will meet after half the expected number of infections have occurred in the trial. The purpose of the DSMB will be to review safety concerns, conduct and review the interim analysis and review external data that may have bearing on the design of or decision to continue the trial. The committee will meet at

further 1 month intervals and provide feedback to the principal investigator; however, the responsibility for the final decision regarding a DSMB-recommended course of action will rest with the principal investigators.

In the event that the outbreak has ended at one or more study sites and the study has not yet been completed, the study should be paused. Efficacy data from the trial must not be prematurely released. In this instance, the DSMB should conduct an interim analysis of the study data to make recommendations whether the study should continue or stop for efficacy, futility or safety. As per World Health Organization policies on data-sharing in a Public Health Emergency, clinical trial outcome data will be shared at the earliest possible opportunity.

7.4 Informing Participants of Amendments After Enrollment

Several participants will have started participation in this study prior to the amendment of May 13th, 2020. Participants already enrolled will be called by their study coordinator to review the amendments and they will be invited for their 1 month study visit at the earliest possibility in the visit window (21 days-30 days) to review and sign the addendum form and then complete additional procedures.

8.0 CLINICAL AND LABORATORY EVALUATIONS

8.1 Clinical Evaluations

All in-person study visits will begin with a COVID-19 symptom screen (see Questionnaire Content document). If the participant fails the symptom screen, a surgical mask should be placed on the participant and full droplet/contact precautions will be followed by study staff (i.e. surgical mask, eye protection, gown, glove). Any participant screening positive for symptoms of COVID-19 will then be directed to the most appropriate place for a confirmatory COVID-19 test (within the department or occupational health as per site institutional guidelines).

For all COVID-19 screen negative participants, study staff will conduct brief interviews to document medical history (baseline comorbidities and medications at day 1 and then new comorbidities, medications, adverse events and side effects at day 30,60,90,120). No physical examinations are required in this study. All WOCP will have a screening urine test at Day 1, 30, 60 and 90. All participants will have a review of a baseline QTc interval measure at Day 1 and a repeat QTc measure at Day 30, 60, 90 and 120. Participants

with a baseline QTc between 430-450 ms will be referred for a repeat QTc measure within 72 hours of commencing study drug.

All side effects will be documented by the study coordinator. The study coordinator has experience in implementing clinical trials of investigational agents and collecting and documenting AEs/SAEs. It is the study physician who will assess severity and relationship to study drug. If the side effect is attributed to the study tablet and has an impact on the participant's functional status (ex. ability to work) or general state of well being, the Study Physician will be responsible for further follow up. Ultimately, the participant will be supported in any decision they make regarding the continuation of study tablets.

If a participant becomes COVID-19 positive at any time in the study, all further in-person clinical evaluations will occur remotely (via telephone call) in light of necessary self-isolation and reducing risk to study staff. They will not have further respiratory specimens or phlebotomy collected while they have symptomatic COVID-19.

Further clinical data will be obtained in weekly emails sent to participants to fill in a short online form including self-report of COVID-19 symptoms/testing/outcomes/time off work, and adherence. Measures of psychological distress will be sent via email in a similar fashion at 1, 60 and 120 days.

Data will be recorded onto standardized electronic forms using the Research Electronic Data Capture system (REDCap) housed on secure servers at the AHRC at St. Michael's Hospital.

8.2 Laboratory Evaluations and Specimen Collection

8.2.1 Overview of Biologic Sample Collection

- Viral detection samples will be self-collected by participants with instructions from study staff (see Section 8.2.1 and 8.2.2)
- Venous blood samples will be collected by study research coordinators. All research coordinators have been trained and are experienced with venipuncture . They have all be trained and will be retrained on PPE donning and doffing as part of study start-up training. This will be kept as part of ISF training log.
- There will be strict observance of IPAC guidelines on interactions with asymptomatic participants (current recommendation: routine precautions) and symptomatic participants (current recommendation: droplet/contact precautions) as outlined in Section 7.2.

- Urine samples will be self-collected by female participants of child bearing age into sterile urine containers for pregnancy testing.

8.2.2 Microbiologic testing for SARS-CoV-2

Specimens for viral RNA testing will be collected at each study visit. Participants will be instructed on self-collection of nasopharyngeal, oropharyngeal, nasal and/or oral swabs or saliva for RT-PCR testing and specimens will undergo biobanking if not tested in real-time for future analysis. The final collection method to be used in the study will be made based on the availability of the collection kits for the study. If oropharyngeal/nasal swabs are not available, saliva will be collected. Self-collection of swabs has been validated in multiple other settings and will allow for reduced exposure to study staff.[30-32] These tests are for the purposes of investigating asymptomatic COVID-19 infection (as per the protocol any symptomatic participants will be tested through the most appropriate institutional methods – ie. occupational health or the ED). Specimens will be extracted using an automated system (easyMAG) and real-time PCR conducted using primers and probes against genes encoding the RNA-dependent RNA polymerase (RdRp) and the envelope (E) protein of COVID-19. Quantitation against a standard curve will be calculated in RNA copies/microliter.

8.2.3 SARS-CoV-2 Serology

Methods for serologic testing of COVID-19 are still in development worldwide at the time of writing, but are rapidly emerging. Participants will undergo venipuncture at baseline, and monthly until and including the 120 day visit and samples processed into plasma for frozen storage by Dr. Kevin Kain's laboratory (co-PI). Frozen samples will be batch-tested in the laboratory of Co-I Mubareka at the conclusion of the study when methods are finalized.

8.2.4 Plaque reduction assay

Participants who become infected will have had venous samples drawn and plasma previously drawn at scheduled monthly visits, collected by research staff and brought according to an approved SOP to the Kain laboratory. It will be frozen at -80C and later tested for SARS-CoV-2 viral plaque reduction assay to determine circulating antiviral activity in infected participants. These studies will be in a BSL3 facility approved for these studies (conducted by Dr. Samira Mubareka). UHN and U of T Biosafety have approved sample handling for this study.

8.2.5 Biomarkers of disease severity

Plasma samples (minimum required volume of 250 μL) collected from participants at study enrolment and scheduled study visits will be processed via a custom multiplex Luminex®-based assays or ELISA to quantify plasma levels of markers of host response

to infection including inflammation, gut-leak, and endothelial activation in the laboratory of Dr. Kevin Kain. Analytes include Angiopoietin (Ang)-1, Ang-2, soluble Tyrosine kinase with immunoglobuline-like and EGF-like domains (sTIE)1, sTIE2, Vascular Endothelial Growth Factor (VEGF), soluble VEGF-receptor1, soluble Endoglin (sEng), soluble Intercellular Adhesion Molecule (sICAM), soluble Tumour Necrosis Factor (sTNF) receptor 2 (sTNFR2), C5a, Chitinase-3-like protein 1 (CHI3L1), C-reactive protein (CRP), Interleukin (IL)-18 binding protein (IL-18BP), IL-6, Zo-1, zonulin, Lipopolysaccharide-binding protein (LBP) ,and Intestinal fatty acid binding protein (I-fabp). Proteins were selected based on evidence of a critical role of in inflammatory pathways that mediate disease severity.

UHN and U of T Biosafety have approved sample handling and the testing procedure for this study.

8.3 Stored Research Specimens and Plans for Possible Future Testing

Any respiratory, serum and/or plasma specimens collected from participants but not analyzed in real-time will be stored in a biobank for potential future testing related to respiratory pathogen diagnostics and inflammatory biomarkers. Consent will be obtained for the storage of these samples for future testing.

8.4 Questionnaires and/or Scales

Questionnaires will be electronically administered weekly through REDCap, which will be emailed to participants directly (consent will be granted for email contact). Those lacking email/internet access may complete questionnaires on paper and/or by interview by study staff (may be done remotely). Then, weekly, all participants will be asked to complete self-report of COVID-19 symptoms/testing/outcomes/time off work, and adherence. For participants with confirmed COVID-19, questions will also include symptom duration, hospitalization, respiratory support and time ineligible/unable to work due to COVID-19. An email reminder will be sent for each questionnaire at day three of each weekly cycle and repeated twice.

The same online system will be used to measure study drug adherence, using a modified version of the ACTG adherence scale.[33,34] This scale was developed by the AIDS Clinical Trials Group to assess adherence to antiretroviral therapy. This scale is brief, easy to use, and was rigorously developed, with high internal consistency and construct validity among adults taking antiretroviral therapy for HIV. Results will be scored according to the included items from this scale.

Finally, at day 1, 60 and 120, participants will also be asked to complete the K10, a short ten-item scale to measure non-specific psychological distress.[28] A standard cutoff score of ≥16 will be used to define short-term psychological distress.

9.0 STUDY PROCEDURES

9.1 Schedule of Events

Table 1: Schedule of Events

	Screening (Day 0)	**Visit 0** Day 1 (±14d)	Home Specimen*1 Day 14	**Visit 1** Day 30 (± 7d)	Home Specimen* 2 Day 44	**Visit 2** Day 60 (±7d)	Home Specimen*3 Day 74	**Visit 3** Day 90 (± 7 d)	Home Specimen*4 Day 104	**Visit 4** Day 120 (± 7 d)
**Visit format: Remote**										
Information video	X									
Self-eligibility assessment	X									
**Visit format: In person**										
Informed consent: individual written consent		X								
Randomization		X								
Dispensation of study drug		X		X		X		X		
Interview by study staff		X		X		X		X		X
Adverse event assessment				X		X		X		X
Self-collected viral detection sample*		X	X	X	X	X	X	X	X	X
Blood for laboratory outcomes		X		X		X		X		X
Urine for pregnancy tests (female participants of reproductive potential)		X		X		X		X		

Evaluation of QT interval		X^^		X		X		X		X
Return Home Specimen										X
**Visit Format: Remote**										
Baseline questionnaire		X								
Weekly visit-specific questionnaire****		X		X		X		X		X
Home specimen reminder**			X		X		X		X	
Short-term psychological assessment***		X				X				X
Unscheduled visits^		X		X		X		X		X

* Depending on availability of viral detection samples supplies, this will be either be a swab (defined in section 8.2.2) or saliva.
** Weekly short electronic questionnaire will be emailed to participants: including new or pending confirmation of COVID-19 results, clinical outcomes of COVID-19, days unable/ineligible to work due to COVID-19 and adherence.
*** An electronic message will be emailed to participants reminding them to collect their viral sample. The email will prompt them to acknowledge that the sample had been collected.
**** These assessment tools will be sent to participants via email to be answered remotely.
^ Study coordinators will be available for unscheduled visits remotely if participants have concerns, problems with study drug, procedures.
^^. This will include either a baseline ECG conducted at the time of enrolment, or review of a resting ECG done within the past 3 months.

Special Schedule Considerations:

If any participant is under quarantine for suspected COVID-19 (ie they are awaiting confirmatory testing results) and, therefore, unable to make a study visit in a visit window, a remote visit will occur and every effort will be made to have an in-person study visit scheduled as soon as they are cleared as negative for COVID-19 for sample collection.

If any participant is confirmed COVID-19 positive during the study, the above scheduled visits will continue *remotely* (telephone) and the weekly email questionnaire will continue. During the time in which they are symptomatic and under isolation, the following events will be suspended: dispensation of drug, self-collected viral detection sample, venous blood draw. COVID-19 positive participants who have recovered and have been deemed safe to return to work by their institution will be asked to return to the above study schedule.

COVID-19 positive participants who have been deemed safe to return to work will be given the option of either a) resuming their blinded study drug and then entering the open-label extension at day 90 as previously scheduled, or b) starting the 30-day open-label extension immediately. The rationale for providing the option to resume blinded study drug is that it remains unknown whether individuals who recover from COVID-19 infection remain susceptible to re-infection, and acquiring COVID-19 does not necessarily mean that the blinded study drug that the participant had been using was not providing partial protection. The rationale for providing the option to skip immediately to the open-label extension is that some participants may understandably prefer to abandon their study drug under these circumstances, and the study has committed to providing every participant with a one-month open-label extension.

9.2 Information and Eligibility Session: (Day 0) - Conducted remotely

Healthcare workers from the participating sites will be invited via email to view an online informational video in which study information, regarding the informed consent document and eligibility criteria will be provided and reviewed. This private YouTube video will not be searchable from within YouTube nor via external search engines (ex. Google). It will only be accessible by the link that will be emailed to hospital employees via their hospital email address. The Department Heads, supervisors, and other persons with authority over the potential participants will not be given access to any information regarding who has or has not accessed the video, or who is or is participating in the study. Access to this informational video will be available to potential participants at UHN and all other

participating sites (SHS, SMH, SHSC) who have been issued the link. The email containing the link to the informational video will also include a Study Fact Sheet as an attachment. Viewing the video or reviewing the Study Fact Sheet does NOT replace written informed consent (see Section 9.3). This approach to the informed consent process is congruent with current physical distancing practices in the case of a public health emergency during COVID-19. Participants willing and who self-screen as eligible will be able to have an individual appointment with study coordinators within 14 days of viewing the informational video in order to undergo the formal eligibility screening and consent process.

9.3 Baseline Visit: (Day 1) Conducted in-person

Participants willing and who self-screen to be potentially eligible will be asked to attend individual appointments with study coordinators within 14 days of information sessions. These baseline visits will be conducted over consecutive days and will allow participants to ask questions about participation and allow the study staff to provide, review and obtain the informed written consent, assess for eligibility, and obtain multiple forms of contact information. All WOCP will have a screening urine pregnancy test for eligibility and all participants will have a baseline QTc interval reviewed and recorded. Written informed consent will be obtained before any study specific activities occur.

Participants who are enrolled at this point will complete the baseline study visit which will include:

- **Randomization and dispensing study drug:** The coordinator must confirm receipt of the package with the participant, and review dosing.
- **Interview:** The coordinator should obtain information from participants to complete the baseline medical history and concomitant medication assessment.
- **Baseline resting ECG:** The coordinator will review a baseline resting ECG and record the QTc interval.
- **Online Questionnaire instructions:** The coordinator should explain to the participant how to access and complete the online questionnaire that will be sent to them via email weekly.
- **Self-collected viral detection sample:** The coordinator should review the printed instructions on how to self-collect, label, date and package the viral detection sample. The participant should then collect and date the sample and package it.
- **Blood for serology and other laboratory markers:** The coordinator should collect one tube of blood (10ml EDTA).
- **Baseline questionnaire:** will be emailed to participant to complete at day 1 (see Questionnaire Content document submitted)

- **Psychological distress questionnaire:** will be emailed to participant to complete at day 1.

9.4 Study Visits 1-4 (Follow-up at Day 30, 60, 90, and 120): Conducted in person.

Participants will be schedule for monthly Study Visit appointments. These are expected to take a maximum of 30 minutes, and in many cases much less. Whenever possible, the study visits will be coordinated with the participant's scheduled ED shift in order to minimize travel and burden on the participant. The components of the Study Visit are as follows:

- **Interview:** The coordinator will review the participant's COVID-19 symptom reporting, their responses to the Adverse Events Questionnaire and Tolerability Questionnaire. The research coordinator will review the responses prior to releasing the next installment of study drug/placebo. If the participant has any concern regarding adverse events or other aspect of the study, they will have the opportunity to discuss these with the research coordinator during the visit.
- **Urine pregnancy test:** for women of childbearing potential.
- **Self-collected viral detection sample:** As above.
- **Blood for serology and other laboratory markers:** As above.
- **QTc interval evaluation:** The QTc will be measured and recorded.
- **Dispense study drug:** The coordinator must confirm receipt of the package with the participant, and review dosing.
- **Psychological distress questionnaire:** will be emailed to participant to complete at day 60 and day 120.

Participants with confirmed COVID-19 will be asked to discontinue their study tablets (see Section 6.7). They will not present for in person visits until they are cleared to return to work.

9.5 Weekly virtual questionnaire (Day 7 to day 120): Conducted remotely

Questionnaires will be administered weekly electronically through REDCap, which will be emailed to participants directly. Self-reported questionnaires will include ascertaining COVID-19 testing underway or completed (with results), adherence scales, and for participants with confirmed COVID-19, questions will also include symptom duration, time off work and hospitalization. An email reminder will be sent for each questionnaire.

9.6 Every Two-Weekly Viral Detection Sample Collection

Participants will be screened for the presence of SARS-CoV-2 every two weeks by the methods described in Section 8.2.2. On the weeks that correspond to the monthly Study Visit, this collection will occur during the Study Visit. During the intervening weeks, participants will self-collect the sample at home and store them in a biosafety bag in their freezer. They will receive an email reminder to collect the sample on these weeks. The participant will return the sample to the study coordinators at their next monthly.

9.7 Early Termination Visit

If a participant chooses to withdraw from the study prematurely after the baseline visit but before the study end, efforts should be made to complete all study activities at the next closest study visit. Participants who discontinue study drug prematurely but consent to still be followed up in the study should follow routine protocol-defined procedures.

9.8 Re-contact of Participants after Trial Termination

Participants will be consented to be re-contacted after trial termination for participation in follow-up studies.

10.0 EVALUATION and REPORTING OF ADVERSE EVENTS

10.1 Adverse Event Reporting

Toxicities will be graded on a 4-point scale according to standard guidelines outlined in the "Division of AIDS Table for Grading the Severity of Adult and Pediatric Adverse Events". In general, grade 1 and 2 events will not be considered adverse events, but details should be documented in the source documents. Stable chronic conditions which are present prior to clinical study entry and do not worsen are not considered adverse events and will be accounted for in the participant's medical history. Participants who have an adverse event (AE) should be followed and treated by the site Investigator until the abnormal parameter or symptom has resolved or stabilized. It is up to the clinician to determine that the AE is either resolved or that is has reached a stable state, after which no further follow-up is necessary. There should also be source documentation to support this determination.

At each contact with the participant, information regarding adverse events will be elicited by appropriate questioning and examinations, and will be recorded immediately on a source document (e.g., progress notes, laboratory reports, survey tools, and data collection tools). The start date, stop date, severity of each reportable event, and the PI's judgment of the AE's relationship to the study medication/intervention will also be recorded.

The Investigator or designee must report any severe adverse event (SAE) occurring in a participant receiving study drug to the Sponsor within 24 hours, even if the SAE is not drug-related. This should be done by telephone and by sending a faxed or emailed copy of the Serious Adverse Event form plus other supporting documentation, as required. All additional follow-up evaluations must also be reported as soon as possible in the same manner. All SAEs will be followed until the Investigator agrees that the event is satisfactorily resolved, becomes chronic, or that no further follow-up is required.

A Serious Adverse Event is defined as an AE meeting one of the following:

- Death during the period of protocol-defined surveillance
- Life Threatening Event (immediate risk of death at the time of the event)
- In-patient hospitalization or prolongation of existing hospitalization during the period of protocol-defined surveillance
- Results in congenital anomaly or birth defect
- Results in a persistent or significant disability/incapacity
- Any other important medical event that may not result in one of the above outcomes may

The intensity (severity) of each adverse/serious event will be graded according to the toxicity table and toxicity guidelines provided by the DAIDS Table for Grading the Severity of Adult and Pediatric Adverse Events. The DAIDS Table will be provided with the Operations Manual.

All SAEs which occur during the course of the study must be reported to the site Project Manager within 24 hours of the site becoming aware of the event. The Project Manager will be responsible for reporting SAEs to Health Canada on behalf of the Sponsor-Investigator.

SAEs will be reported to:

University Health Network
Attention: Rosemarie J. Clarke RN, MHM, BScN, CHE, CCRP
Clinical Research Nurse Manager & Project Manager

University Health Network/Toronto General Hospital
585 University Avenue, 13 North
Toronto, ON M5G 2N2

Phone: 416-340-4800 ext. 2723
Fax: 416-340-9105
Email: rosemarie.clarke@uhn.ca

Study medication will be discontinued in the following instances: unacceptable toxicity, or toxicity that, in the judgment of the investigator, compromises the ability to continue study-specific procedures or is considered to not be in the subject's best interest.

11.0 STATISTICAL CONSIDERATIONS

11.1 General Study Design

This is a double-blinded, individually randomized, parallel-group randomized placebo controlled clinical trial.

11.2 Sample Size Considerations/Justification

The 3-month risk of infection in healthcare workers not treated with prophylaxis was assumed to be 10%. This corresponds to a hazard of 0.035 infections per month under the assumption of exponentially-distributed time to infection. Reducing 3-month risk to ~6% was judged to be both feasible and clinically important; this reduction corresponds to a hazard ratio of 0.588. With a one-sided type I error rate of 5%, to have 80% power, to detect this hazard ratio, a total of 92 events are needed. Using the event rates above and exactly 3 months + 14 days planned follow-up for each participant, a total of 988 participants need to be randomized. These calculations account for the single interim analysis described below.

11.3 Endpoints/Outcome Measures

11.3.1 Analysis of Primary Outcome Measures

The primary outcome comprises a microbiologically confirmed COVID-19 infection and the date of the confirmatory test. At the date of an analysis (interim or final), each participant will have either an infection and date of infection, or a known uninfected status and time since randomization (censoring date). The rates of the outcome will be

compared between randomized groups using a Cox proportional hazards model with stratification by site (used in the stratified randomization) and inclusion of job class as a covariate. As there are too many distinct job classes in the inclusion criteria for them all to be included in the model, they will be grouped before any analysis begins. The grouping will combine classes of job deemed to be at similar levels of exposure, while keeping groups a reasonable size. If there is heterogeneity in risk by job class, inclusion of this factor in the analyses increases power with compromising type I error. See section 9.5 for details on inference at the interim and final analyses. A plot of cumulative incidence of infection over the 104-day study period will be made for each group and the overall rates of infection in each study arm will be calculated. All primary analyses will be by intention-to-treat. While loss to follow-up is expected to be low, if more than 5% of participants withdraw, missing data will be imputed under a variety of assumptions, to assess the robustness of our findings.

11.3.2 Analysis of Secondary Outcome Measures

Adverse events will be tabulated by study arm. The percentages of participants experiencing each type of AE, or any AE will be calculated by severity level. The rate per 100 person-days will also be calculated by AE and severity level. If warranted, AEs will be compared between groups using the methods for the primary analysis. **Symptom duration,** **duration of hospitalization due to COVD-19 disease, days of respiratory failure requiring ventilatory support attributable,** and **number of days ineligible/unable to work due to COVID-19** will be summarized within group by the median and interquartile range as well as the mean. As the majority of participants will have zero for all of these outcomes, a bootstrap hypothesis test, with stratification by site, will be used to test the hypothesis of no treatment effect. A 95% confidence interval for the mean difference in days will also be computed using the bootstrap. Mortality is expected to be very low, so will simply be summarized by a count and percentage in each arm. The relative risk of mortality by 90 days will be calculated if feasible. **Seropositivity** will be analyzed by the same approach described for the primary outcome. **Short-term psychological impact of exposure to COVID-19** as measured by the K10 will be summarized by group at each of the three time points (day 1, 60, and 120), using the mean and standard deviation as well as the percentages with scores at or above the standard cutoff score of 16. As the trial is blinded, there is no expectation of a direct effect of treatment on the K10. Those who become infected may have higher scores, so results will also be presented according to the previous occurrence of an infection. The primary comparison of this outcome will use a linear model to estimate the effect on 60-day values of site, study arm, and job class, using the day 1 value as a covariate. The relative risks of exceeding the threshold at 60 days for these same variables will be estimated in a log-binomial model with the same covariate. The change in K10 between days 60 and 120

will be estimated to assess whether there is a reduction in psychological impact once participants know they are on prophylaxis with an active drug.

11.3.3 Interim Analysis

One interim analysis of the primary outcome is planned when half the expected number of events (46/92) has occurred. The table below shows the properties of this design. At the first interim analysis, a Z-statistic (-log(HR)/(Standard Error)) larger than 2.67 will be taken as evidence of efficacy; this is the same as checking whether the one-sided p-value is < 0.0038. If the true hazard ratio is 0.588 (as in the sample size calculation), there is a 19% chance of stopping for efficacy at this interim analysis. The trial may stop for futility (with the recommendation coming from the DSMC, as this is a non-binding futility boundary) if the Z-statistic is less than -0.01, which corresponds to the one-sided p-value being > 0.51. If the true HR is 1.0, there is a 49% chance that the interim analysis will cross this futility boundary. If the trial does not stop for futility or efficacy, the final analysis will take place at the earlier of the date that all participants have reached the end of the 104-day randomized or when 92 events have occurred. This final analysis will declare superiority of HCQ over placebo if the Z-statistic is > 1.65 (or the one-sided p-value is < 0.049). With 92 total events, power is 80% against HR=0.588 and type I error is 5% across the whole trial. One-sided testing is justified here to increase power, as there is no interest in concluding that HCQ is inferior to placebo. A finding other than HCQ being superior will lead to the same decision not to use it.

Analysis	Value	Efficacy	Futility
IA 1: 50%	Z	2.6700	-0.0142
N: 988	p (1-sided)	0.0038	0.5057
Events: 46	HR at bound	0.4542	1.0042
Month: 1.7	P(Cross) if HR=1	0.0038	0.4943
	P(Cross) if HR=0.59	0.1864	0.0365
Final	Z	1.6549	1.6549
N: 988	p (1-sided)	0.0490	0.0490
Events: 92	HR at bound	0.7076	0.7076
Month: 3.5	P(Cross) if HR=1	0.0489	0.9511
	P(Cross) if HR=0.59	0.8000	0.2000

At the final analysis, the hazard ratio, its 90.2% confidence interval will be calculated, as the upper limit of the 90.2% CI will lie below 1 if the null hypothesis that HR=1 is rejected.

12.0 STUDY ETHICAL CONSIDERATIONS

12.1 Ethical Conduct of the Study

This study will be conducted in accordance with the ICH-GCP Guidelines and the principles in the Declaration of Helsinki. Investigators will be thoroughly familiar with the appropriate use of the study drug as described in the protocol and product monograph.

12.2 Informed Consent

All participants will be given detailed oral and written information about the study. Consent forms describing in detail the study medication and risks will be given to each participant and written documentation of informed consent is required prior to starting study medication. Participants must sign an informed consent document that has been approved by a participating centre's REB prior to any procedures being done specifically for the trial. Each participant should have sufficient opportunity to discuss the study, have all their questions addressed and consider the information in the consent process prior to agreeing to participate. Participants may withdraw consent at any time during the course of the study without prejudice. The informed consent form will be signed and dated by the participant and the investigator or delegate. The original signed informed consent form will be retained in the participant's study files and a copy will be provided to the participant.

The informed consent process must be conducted, and form signed before the participant undergoes any screening procedures that are performed solely for the purpose of determining eligibility for the study.

12.3 Confidentiality

All participant-related information including consent forms, questionnaires, laboratory specimens, evaluation forms, reports, etc. will be kept strictly confidential. All records will be kept in a secure, locked location and only accessible to research staff. Participants will be identified only by means of a coded number specific to each participant. All computerized databases will identify participants by numeric codes only, and will be password protected.

Upon request, and in the presence of the investigator or his/her representative, participant study records will be made available to the study sponsor, monitoring groups representative of the study sponsor, representatives of funding groups, and applicable regulatory agencies for the purpose of verification of clinical trial procedures and/or data, as is permissible by local regulations.

12.4 Research Ethics Board

The REB will review all appropriate study documentation to safeguard the rights, safety, and well-being of the participants. The study will be conducted only at sites where ethics approval has been obtained. A copy of the protocol (including protocol amendments), all versions of informed consent forms, other information to be completed by participants such as survey instruments or questionnaires, and any proposed advertising/recruitment materials must be reviewed and approved by the REB. The investigator will be responsible for obtaining REB approval of the annual Continuing Review throughout the duration of the study. The investigator will notify the REB of serious adverse events as applicable. The investigator will seek prior ethics approval for any protocol deviations except when the change is intended to eliminate an immediate hazard to participants. In this case, the protocol deviation will be promptly reported.

13.0 General Trial Conduct Considerations

13.1 Adherence to Protocol

13.1.1 Protocol Amendments

All protocol amendments will be reviewed and approved and if applicable submitted to the applicable regulatory agencies for prior approval or notification. The Investigator must sign and date the amendment prior to implementation. All protocol amendments must also be submitted to the ethics committee.

13.1.2 Protocol Deviations

No deviations from this protocol will be permitted without the prior written approval of the Sponsor-Investigator, except when the modification is needed to eliminate an immediate hazard or hazards to participants. Any deviations that may affect a participant’s treatment or informed consent, especially those increasing potential risks, must receive prior approval from the REB unless performed to remove an immediate safety risk to the participants. In this case it will be reported to the REB and the Sponsor-Investigator immediately thereafter. Any departures from the protocol must be documented.

13.2 Monitoring & Auditing

13.2.1 Study Monitoring

Each study site agrees to allow representatives of the trial data management centre (Applied Health Research Centre of St. Michael's Hospital; AHRC) to have direct access to the study records from those participants enrolled in the clinical study as well as Investigational Product accountability records, in order to conduct remote risk-based monitoring of this trial. The proposed remote monitoring scheme will be composed of:

1) Centralized review of essential study document at all participating sites related to participant protection, such as ICF signature pages, GCP and protocol training records, Delegation log, CV and medical license of investigators and Protocol signature page
2) Targeted source data verification (SDV) of eCRF data will be performed on 10% of participants chosen at random. Only critical data variables identified through risk assessment which are programmed into the EDC system will be source verified.

AHRC will provide sites with the SDV Tool, which is a one page document listing key variables for targeted SDV. Sites will be asked to complete the SDV Tool and send it to the central coordination team, along with de-identified source documents for selected variables listed on the SDV Tool via a dedicated end-to-end secure internet portal. Instructions will be provided to sites on how to complete the SDV tool, de-identify source documents, and send these documents to central coordination team using the secure portal.

Upon receipt of the completed SDV Tool and appropriate source documents, the central coordination team will review these documents to ensure that the data is consistent with the data entered on eCRF. The review will include checking the eCRF entries for accuracy and completeness against source documents. AHRC will maintain a monitoring log of all participants for whom source documents are requested, received, and verified. Variables will be marked as "verified" in the monitoring log once the review is complete. Urgent issues will be communicated on an ongoing basis as needed with the PIs/Sponsor.

If errors or inconsistencies are noted, a follow up email will be sent to the site's Principal Investigator and Primary Study Coordinator. The follow up email will include a summary of the issues identified, outline of any corrective actions and/or request an explanation, and a timeline for resolution.

13.3 Record Keeping

13.3.1 Data Collection
The Investigator must maintain detailed records on all study participants. Data for this study will be recorded in the participant's chart and entered into case report forms (CRFs). Copies of CRFs will remain at the clinical site at the conclusion of the study.

13.3.2 Data Corrections
Any corrections of data entered on source documents at the study site should be crossed out with a single horizontal line, initialed and dated.

13.3.3 Source Documents
The Investigator must maintain adequate and accurate source documents upon which CRFs for each participant are based. They are to be separate and distinct from CRFs except for cases in which the Sponsor-Investigator has pre-determined that direct data entry into specified pages of the participant's CRF is appropriate. These records should include detailed notes on:

- Oral and written communication with participant regarding the study treatment (risks/benefits)
- Participation in trial and signed and dated informed consent forms
- Inclusion and exclusion criteria details
- Visit dates
- Adverse events and concomitant medication
- Participant's exposure to any concomitant therapy
- Reason for premature discontinuation (if applicable)
- Enrollment number
- Adherence to the study protocol and protocol deviation information

13.3.4 Data Management
Data management responsibilities for this trial will be assumed by the AHRC. Instructions concerning the recording of study data on CRFs will be provided in a comprehensive study Operations Manual. Each study site is responsible for submitting the data in a timely fashion. Detailed aspects of data handling will be described in the Data Management Plan.

13.3.5 Record Retention
The Investigator will maintain all study records according to the ICH-GCP and applicable Health Canada regulatory requirements. Records will be retained for 25 years, in accordance with applicable Health Canada regulatory requirements. If the Investigator withdraws from the responsibility of keeping the study records, custody must be transferred to a person willing to accept the responsibility and the Sponsor-Investigator

notified. The Investigator should ensure that no destruction of medical records occurs without the written approval of the Sponsor-Investigator.

13.4 Steering Committee

The steering committee (SC) will include the two Co-Principal Investigators (Landes, Kain), together with other pre-selected investigators with appropriate methodologic and content expertise. The SC will meet bi-weekly to discuss epidemic trends/recruitment, ethical issues, and emerging scientific or logistical issues. The SC will advise the Co-PIs on relevant trial decisions, but final decision-making authority will rest with the Co-Principal Investigators.

14.0 Disclosure and Publication Policy

14.1 Publication of Study Results

Following completion of the study, the lead Principal Investigator is expected to publish the results of the primary and secondary analyses from this trial, in peer-reviewed scientific journals. A detailed authorship policy will be developed and agreed upon by all investigators to determine how best to fairly acknowledge the contributions of relevant parties.

14.2 Data Sharing for Secondary Research

Data from this study may be used for secondary research. All of the individual participant data collected during the trial will be made available after de-identification through expert determination. These data will be made available as soon as possible following publication, with no end date, as part of data sharing requirements from journals and funding agencies, and in the spirit of open data access.

14.3 Conflict of interest

The independence of this study from any actual or perceived influence, such as by the pharmaceutical industry, is critical. Therefore, any actual conflict of interest of persons who have a role in the design, conduct, analysis, publication, or any aspect of this trial will be disclosed and managed. Furthermore, persons who have a perceived conflict of interest will be required to have such conflicts managed in a way that is appropriate to their participation in the design and conduct of this trial.

The Study Sponsors, study sites, and all protocol contributors have no conflicts of interest. The funding bodies have had / will have no role in the design of the study, data collection, analysis, interpretation, or dissemination of study findings to either journals, general public or scientific meetings.

15.0 REFERENCES

1. “WHO Director-General’s opening remarking at the media briefing on COVID-10.” World Health Organization (WHO) [Press release]. March 11, 2020. [Accessed Mar 21, 2020].

2. World Health Organization (WHO). 2020. Coronavirus disease (COVID-19) outbreak situation. [ONLINE] Available at: https://www.who.int/emergencies/diseases/novel-coronavirus-2019. [Accessed Mar 22, 2020].

3. Bein S. “Coronavirus Update: Provinces threaten fines, arrests to enforce isolation; Vancouver bans most visitors to long-term care homes.” The Globe and Mail. Mar 21, 2020. Available at: https://www.theglobeandmail.com/canada/article-coronavirus-update-provinces-threaten-fines-arrests-to-enforce/. [Accessed Mar 22, 2020].

4. McGoogan JM, Wu Z. Characteristics of and Important Lessons from the Coronavirus Disease 2019 (COVID-19) Outbreak in China: Summary of a Report of 72,314 Cases from the Chinese Center for Disease Control and Prevention. JAMA Network. JAMA. Published online February 24, 2020. doi:10.1001/jama.2020.2648.

5. Gruppo Italiano per la Medicina Basata sulle Evidenze (GRIBE). 2020. Online la dataroom GIMBE sull’emergenza COVID-10 a disposizione di istituzoni e mezzi di informazione. Available at: https://www.gimbe.org/pagine/341/it/comunicati-stampa. [Accessed Mar 22, 2020].

6. Remuzzi A, Remuzz Gi. COVID-19 And Italy: What Next?. The Lancet. Elsevier BV, doi:10.1016/s0140-6736(20)30627-9.

7. Gawande A. “Keeping the Coronavirus from Infecting Health-Care Workers.” The New Yorker. Mar 21, 2020. Available at: https://www.newyorker.com/news/news-desk/keeping-the-coronavirus-from-infecting-health-care-workers. [Accessed Mar 22, 2020].

8. Kain T, Fowler R. Preparing intensive care for the next pandemic influenza. Critical Care. 2019;23(337):10.1186/s13054-019-2616-1.

9. Rothe C, Schunk M, Sothmann P, Bretzel G, Froeschl G, et al. Transmission of 2019-nCoV Infection from an Asymptomatic Contact in Germany. N Engl J Med. 2020;382:10.

10. Li R, Pei S, Chen B, Song Y, Shaman J, et al. Substantial undocumented infection facilitates the rapid dissemination of novel coronavirus (SARS-CoV2). Science. 2020:10.1126/science.abb3221.

11. Vincent, M.J., Bergeron, E., Benjannet, S. et al. Chloroquine is a potent inhibitor of SARS coronavirus infection and spread. Virol J 2, 69 (2005). https://doi.org/10.1186/1743-422X-2-69.

12. Savarino A, Boelaert JR, Cassone A, Majori G, and Cauda R. Effects of chloroquine on viral infections: an old drug against today's diseases? The Lancet: Infectious Diseases. 2003;3:722-727.

13. Wang, M., Cao, R., Zhang, L. et al. Remdesivir and chloroquine effectively inhibit the recently emerged novel coronavirus (2019-nCoV) in vitro. Cell Res 30, 269–271 (2020). https://doi.org/10.1038/s41422-020-0282-0.

14. Gao J, Tian Z, Yang X. Breakthrough: Chloroquine phosphate has shown apparent efficacy in treatment of COVID-19 associated pneumonia in clinical studies. Biosci Trends. 2020;14(1):72-73.

15. Zhonghu J, He H, Hu X, and Za Z. Multicenter collaboration group of Department of Science and Technology of Guangdong Province and Health Commission of Guangdong Province for chloroquine in the treatment of novel coronavirus pneumonia. Expert consensus on chloroquine phosphate for the treatment of novel coronavirus pneumonia. 2020;43(3):185-188.

16. Biot C, Daher W, Chavain N, Fandeur T, Khalife J, Dive D, et al. Design and synthesis of hydroxyferroquine derivatives with antimalarial and antiviral activities. J Med Chem. 2006;49:2845-2849.

17. Marmor MF, Kellner U, Lai TY, Melles RB, Mieler WF; American Academy of Ophthalmology. Recommendations on Screening for Chloroquine and Hydroxychloroquine Retinopathy (2016 Revision). Ophthalmology. 2016;123(6):1386-1394.

18. Yao X, Ye F, Zhang M, Cui C, Huang B, Niu P, et al. In Vitro Antiviral Activity and Projection of Optimized Dosing Design of Hydroxychloroquine for the Treatment of Severe Acute Respiratory Syndrome Coronavirus 2 (SARS-CoV-2). Clin Infect Dis. 2020. doi:10.1093/cid/ciaa237. [Epub ahead of print].

19. Gautret et al. Hydroxychloroquine and azithromycin as a treatment of COVID-19: results of an open-label non-randomized clinical trial. International Journal of Antimicrobial Agents. [In Press Mar 17, 2020]. 2020;doi:10.1016/j.ijantimicag.2020.105949

20. Product Monograph: Apo-Hydroxquine. Products.Apotex.Ca, https://www1.apotex.com/products/ca/downloads/en/di/0097_pil.pdf. Accessed 25 Mar 2020.

21. Bernstein HN. Ocular safety of hydroxychloroquine. Ann Ophthalmol. 1991;23:292–96.

22. Bernstein HN. Ophthalmologic considerations and testing in patients receiving long-term antimalarial therapy. Am J Med. 1983;75:25–34.

23. Herman K, Leys A, Spileers W. (Hydroxy)- chloroquine retinal toxicity: two case reports and safety guidelines. Bull Soc Belge Ophtalmol. 2002;284:21–29.

24. Klinger G, Morad Y, Westall CA, et al. Ocular toxicity and antenatal exposure to chloroquine or hydroxychloroquine for rheumatic diseases. Lancet. 2001;358:813–14.

25. Lazovski J, Losso M, Krohmal B, Emanuel E. J, Grady C, & Wendler D. (2009). Benefits and burdens of participation in a longitudinal clinical trial. Journal of empirical research on human research ethics, 4(3), 89–97. https://doi.org/10.1525/jer.2009.4.3.89

26. Division of AIDS (DAIDS) Table for Grading the Severity of Adult and Pediatric Adverse Events, Version 2.0, November 2014. Division of AIDS, National Institute of Allergy and Infectious Diseases, National Institutes of Health, US Department of Health and Human Services; 2014.

27. Furukawa TA, Kessler RC, Slade T, Andrews G. The performance of the K6 and K10 screening scales for psychological distress in the Australian National Survey of Mental Health and Well-Being. Psychol Med 2003;33:357-62. doi: 10.1017/s0033291702006700.

28. Kessler RC, Andrews G, Colpe LJ, et al. Short screening scales to monitor population prevalences and trends in non-specific psychological distress. Psychol Med 2002;32:959-76. doi: 10.1017/s0033291702006074.

29. Sperber, K., Hom, C., Chao, C.P. et al. Systematic review of hydroxychloroquine use in pregnant patients with autoimmune diseases. Pediatr Rheumatol 7, 9 (2009). https://doi.org/10.1186/1546-0096-7-9

30. Larios OE, Coleman BL, Drews SJ, et al. Self-collected mid-turbinate swabs for the detection of respiratory viruses in adults with acute respiratory illnesses. PLoS One 2011;6:e21335. doi: 10.1371/journal.pone.0021335. Epub 2011 Jun 23.

31. Granados A, Quach S, McGeer A, Gubbay JB, Kwong JC. Detecting and quantifying influenza virus with self- versus investigator-collected mid-turbinate nasal swabs. J Med Virol 2017;89:1295-9. doi: 10.002/jmv.24753. Epub 2017 Feb 27.

32. Corman VM, Landt O, Kaiser M, et al. Detection of 2019 novel coronavirus (2019-nCoV) by real-time RT-PCR. Euro Surveill 2020;25(3).10.2807/1560-7917.ES.2020.25.3.2000045.

33. Reynolds NR, Sun J, Nagaraja HN, Gifford AL, Wu AW, Chesney MA. Optimizing measurement of self-reported adherence with the ACTG Adherence Questionnaire: a cross-protocol analysis. J Acquir Immune Defic Syndr 2007;46:402-9. doi: 10.1097/qai.0b013e318158a44f.

34. Reynolds NR, Sun J, Nagaraja HN, Gifford AL, Wu AW, Chesney MA. Optimizing measurement of self-reported adherence with the ACTG Adherence Questionnaire: a cross-protocol analysis. J Acquir Immune Defic Syndr 2007;46:402-9.

16.0 APPENDICES

16.1 Appendix 1: Patient Consent Document

Informed Consent Form for Participation in a Research Study

Study Title: Protecting Frontline Health Care Workers from COVID-19 with Hydroxychloroquine Pre-exposure Prophylaxis: A Randomized, Placebo-controlled Multi-Site Trial in Toronto, Canada (HEROS Trial)

Study Doctor: insert name, department and telephone or pager number

Sponsor: University Health Network (UHN)
Funders: National Research Council Canada (NRC); UHN Foundation; Study drug provided by Apotex.

Emergency Contact Number (24 hours / 7 days a week): ____________________________

INTRODUCTION

You are being invited to participate in a type of research study called a randomized controlled trial (RCT). You are invited to participate in this trial because you are a front line health care worker (HCW) with high risk of exposure to patients with suspected or confirmed COVID-19.

This consent form provides you with information to help you make an informed choice. Please read this document carefully and ask any questions you may have. All your questions should be answered to your satisfaction before you decide whether to participate in this research study.

The study staff will tell you about the study timelines for making your decision.

Taking part in this study is voluntary. You have the option to not participate at all, or you may choose to leave the study at any time. Whatever you choose, it will not affect the usual medical care that you would receive outside the study. Further, participation or non-participation in this study will not in any way affect your employment. If you have any questions about this you can ask the study team.

IS THERE A CONFLICT OF INTEREST?
There are no conflicts of interest to declare related to this study.

WHAT IS THE BACKGROUND INFORMATION FOR THIS STUDY?

On March 11th, 2020, the World Health Organization (WHO) declared coronavirus disease (COVID-19) caused by severe acute respiratory virus coronavirus 2 (SARS-CoV-2) a pandemic.i Even with current moderate interventions imposed, estimates predict that 10-18% of the Canadian population will be infected with the virus by its peak in July.ii Health care workers (HCWs) remain critical in responding to this pandemic, and yet they are among the most vulnerable to being repeatedly exposed to the virus. It is in our collective interest to protect HCWs from infection such that they may be able to continue to work and provide essential health services to the Canadian population. Hand hygiene and correct donning and doffing of Personal Protective Equipment (PPE) are evidence-based ways to prevent the spread of COVID-19 to HCWs in a healthcare setting.iii A pre-exposure prophylaxis (PrEP) strategy using a safe, effective, and available therapy with activity against SARS-CoV-2 could further reduce HCW risk of infection.

During a pandemic, when there is insufficient time to develop new therapies against a novel pathogen, repurposing existing drugs with known safety and tolerability profiles offers a potentially rapid means to increase available therapeutics against the new threat. In the case of COVID-19, two candidate drugs, chloroquine (CQ) and its derivative hydroxychloroquine (HCQ), have shown some promise. Originally an anti-malarial medication, CQ exerts direct antiviral effects by inhibiting the replication of SARS-CoV-2. HCQ has also been in shown to have more anti-SARS-CoV-2 activity than CQ in vitro.iv It has a better safety profile than CQ (during long term use), and allows a higher daily dose,v with fewer concerns of drug-drug interactions.vi

To our knowledge, there is currently no clinical evidence that HCQ is effective for the prevention or treatment of COVID-19 disease, but preliminary laboratory testing suggests that it may have potential. Rigorous testing of HCQ for this purpose is critical at this time. We therefore aim to conduct the first randomized placebo-controlled trial of HCQ to prevent COVID-19 infections in health care workers with high risk of SARS-CoV-2 exposure, as we prepare for escalating rates of COVID-19 in our hospitals in Toronto, Canada.

Health Canada, the regulatory body that oversees the use of drugs in Canada, has not approved the sale or use of HCQ for prophylaxis or treatment of COVID-19 disease. Health Canada has allowed HCQ to be used in this study.

WHY IS THIS STUDY BEING DONE?

The purpose of this study is to evaluate whether HCQ – taken as pre-exposure prophylaxis and dosed at 400 mg once a day – can reduce COVID-19 infections amongst high risk HCWs during the current pandemic.

To do this, some of the participants in this study will receive HCQ, and others will receive a placebo (a substance that looks like HCQ but does not have any active or medicinal ingredients) for 90 days. The placebo in this study is not intended to have any effect on

COVID-19 disease. A placebo is used to make the results of the study more reliable. At 90 days after enrolment, all participants will be offered 30 days of HCQ in what is called an open-label extension phase of the trial. The open-label extension phase will help us further assess adherence and safety of HCQ. This part of the study is optional, and you will be asked if you wish to participate in the open-label extension phase at the end of this consent form.

WHAT OTHER CHOICES ARE THERE?

To date, there is no evidence that any drug is effective as prophylaxis for COVID-19 and thus, there are no other options for pre-exposure prophylaxis. If you do not wish to participate, you will continue your current practice for preventing infection which includes the correct use of PPE, handwashing and following appropriate infection prevention procedures within your department.

HOW MANY PEOPLE WILL TAKE PART IN THIS STUDY?

It is anticipated that 988 people will take part in this study, from multiple research sites located in Canada.

This study includes a three-month period in which you will be randomized to either the placebo or intervention group followed by one month of the open-label extension. We will complete the final analysis of the study in month four regarding the effect of the study and these results should be known between months 4-6.

WHAT WILL HAPPEN DURING THIS STUDY?

If you decide to participate in this study, you will be "randomized" into one of the groups described below. Randomization means that you are put into a group by chance (like flipping a coin). There is no way to predict which group you will be assigned to. You will a 50% chance of being placed in either group. Neither you, the study staff, nor the study doctors can choose what group you will be in.

This is a double-blind study, which means that neither you, the study doctors, the study staff, nor any other of your health care providers will know which group you are in. Your group assignment can be identified if medically necessary. Requests to reveal your assignment for your information or participation in other research studies will not be considered until this study has been completed and the results are known.

If you agree to take part in this study, you will be randomized to one of the following groups for three months:

Group 1 (Experimental intervention): You will be given HCQ 400 mg to take by mouth once daily for 90 days.

Group 2 (Non-Experimental Intervention): You will be given a placebo that closely resembles the study drug to take by mouth once daily for 90 days.

If you agree to take part in the open-label extension period, at 90 days you will be given HCQ 400 mg to take by mouth once daily for thirty days.

WHAT ELSE DO I NEED TO KNOW ABOUT THE STUDY INTERVENTION?

If you have side effects while you are in this study, the study doctor may decide with you to stop the intervention. There are no known treatments or drugs approved for COVID-19 prophylaxis; however, as part of this study, you will be asked not to take any other experimental treatments as prophylaxis for COVID-19, including either CQ or HCQ. These will be discussed at the time of eligibility.

WHAT ARE THE STUDY PROCEDURES?

Non-Experimental Procedures

You will be asked to have an enrollment visit, 3 follow-up monthly visits as well as an exit visit. At each of these visits, we will collect a venous blood sample and a viral detection sample and will obtain an ECG. These visits should take approximately 15-30 minutes. If samples are not processed during the time of the study, they will be banked for future testing. This banking is optional and you will be asked if you agree to allow your samples to be stored and used for other research at the end of this form.

The following specific tests will be done during these visits as part of this study:
Venous blood draw: Blood samples will be taken by inserting a needle into a vein in your arm. Five samples in total will be taken: at enrollment, day 30, day 60 and day 90, and at exit. One vial of blood (10 mLs) for isolation of plasma will be collected. These samples will be sent to a laboratory at the UHN where they will be stored and examined at a future date.
Viral detection specimen: This will be either a swab from the front of your nose (mid-turbinate), your throat (pharyngeal), the back of your nose (naso-pharyngeal) or a saliva sample. These will be collected by you, with instructions from the study coordinator at the time of your monthly visit. You will also be asked to do up to 7 of these collections at home at defined intervals and keep them in your freezer until the last visit where you will return them to the study site. When returned to the study site, these specimens will be stored in a lab for testing at a future date (i.e. results will not be available in real-time).
ECG: a heart rhythm measurement will be done at every study visit.

The above non-experimental procedures will continue through the open-label extension to day 120 should you agree to participation.

Questionnaires

You will be sent an electronic questionnaire on your day of enrollment. Subsequently, a questionnaire will be emailed to you every week. The purpose of the questionnaire is to understand symptoms you may be having, record any missed medication doses or difficulties you are having taking the medication, and ask about any testing for COVID-19 that may be happening for you if you meet the Public Health or your Hospital's Occupational Health criteria. Each questionnaire will take about 5 minutes to complete.
Three times over the course of the study you will be emailed a secure online survey regarding your levels of anxiety.

The above questionnaires will continue through the open-label extension as above if you agree to participate.

The information you provide is for research purposes only. Some of the questions are personal. You can choose not to answer questions if you wish.

Your responses on the questionnaires will not be reviewed by your health care team or study team. If you wish them to know this information please bring it to their attention.

Please note, all further description of study information for the participant below relates to both the randomization phase of the study and the open-label extension should you consent to participate in the open-label extension.

HOW WILL THE SAMPLES BE IDENTIFIED?

To protect your privacy, your samples will be labelled only with your study identification number and the date of collection. Your name or other personal identifying information will not appear on the sample.

Despite protections being in place, there is a risk of unintentional release of information.

CAN I WITHDRAW THESE SAMPLES?

If you no longer want your samples to be used in this research, you should tell the Sponsor-Investigator, who will ensure the samples are destroyed. However, once the samples are analyzed and the study database anonymized, it will not possible to remove your individual data from aggregate analyses.

OPTIONAL RESEARCH

The Researchers doing this study may be interested in doing additional follow-up research relevant to the diagnosis, management or outcomes of COVID-19 or other acute respiratory illnesses. You will be asked at the end of this form if you agree to having unused samples stored and used for future research. You will also be asked if you agree to have your data stored and used for future research. Additionally, we will ask you at the end of this form for permission to contact you in the future to offer participation in additional studies.

WHAT ARE THE RESPONSIBILITIES OF STUDY PARTICIPANTS?

If you choose to participate in this study, you will be expected to:
Follow Public Health and your Occupational Health department's guidelines for presenting to work, specifically not coming to work if you are sick or feeling unwell.
Follow Public Health and your Occupational Health department's guidelines for COVID-19 testing
Tell the study doctors about your current medical conditions;
Tell the study doctors about all prescription and non-prescription medications and supplements, including vitamins and herbals you are taking, and check with the study doctor before starting, stopping or changing any of these. This is for your safety as these may interact with the intervention that you receive in this study.
Tell the researchers or study doctor if you are thinking about participating in another research study
Return any unused study medication.
Tell the researchers or study doctor if you become pregnant or father a child while participating on this study
The study drug (either placebo or HCQ) is for you alone, and must not be shared with others. If someone accidentally takes 2 or less doses of your study drug they should inform the Sponsor-Investigator/Emergency Contact. If they take more than 2 doses also call the Sponsor-Investigator/Emergency Contact and proceed to the nearest emergency department.
Avoid eating grapefruit or drinking grapefruit juice as it may increase the risk of having side effects.

HOW LONG WILL PARTICIPANTS BE IN THE STUDY?

The study intervention will last for four months.

You will be asked to come back to the designated site visit room every 30 days after the enrollment visit for four visits (30, 60, 90 & 120 days).

No matter which group you are randomized to, and even if you stop the study intervention early, we would like to keep track of your health for 120 days after you enter the study. We would do this by having you come back to the designated site visit room at the hospital or having someone from this study team call you to see how you are doing.

If you miss a study visit or are not completing surveys for two weeks in a row the study team will call you to follow-up. If the team is unable to reach you in a reasonable amount of time, they will call your alternative contact. We will ask for your consent to do this at the end of this form.

CAN PARTICIPANTS CHOOSE TO LEAVE THE STUDY?

You can choose to end your participation in this research (called withdrawal) at any time without having to provide a reason. If you choose to withdraw from the study, you are encouraged to contact the study doctor or study staff.

You may be asked questions about your experience with the study intervention.

You may withdraw your permission to use information that was collected about you for this study at any time by letting the study doctor know. However, this would also mean that you withdraw from the study.

Participation or withdrawal from the study will not affect your employment.

CAN PARTICIPATION IN THIS STUDY END EARLY?

The study doctor may stop your participation in the study early, and without your consent, for reasons such as:
You are unable to tolerate the study intervention
You are unable to complete all required study procedures
New information shows that the study intervention is no longer in your best interest
The study doctor no longer feels this is the best option for you
The Sponsor-Investigator decides to stop the study
Health Canada or a research ethics board withdraw permission for this study to continue
Your group assignment becomes known to you or others (like the study doctor or study staff)

If this happens, it may mean that you would not receive the study intervention for the full period described in this consent form.

If you are removed from this study, the study doctor will discuss the reasons with you and plans will be made for your continued care outside of the study.

WHAT ARE THE RISKS OR HARMS OF PARTICIPATING IN THIS STUDY?

You may experience side effects from participating in this study. The study drug is widely used and the expected side effects are listed below. You should discuss side effects with the study doctor.

The study doctor will watch you closely to see if you have side effects. When possible, other medicine will be given to you to make side effects less serious and more tolerable. Many side effects go away shortly after the study intervention is stopped, but in some cases side effects can be serious, long-lasting, or permanent.

Risks and side effects related to the experimental intervention HCQ we are studying include:

Very Common: (>10%): Abdominal pain, nausea

Common: (1-10%): Blurring of vision, diarrhea, vomiting, anorexia, headache, affect lability, skin rash/itchiness

Uncommon: <1%: Vertigo or dizziness, tinnitus or ringing in your ears, maculopathies or changes in your vision (in early form is reversible), abnormal liver function tests, nervousness, pigmentary changes in skin or hair.

Not known (event rate): blood disorders, cardiomyopathy, prolonged QTC, urticarial or hives, angioedema or swollen tongue/lips, bronchospasm, myopathy.

Serious side effects are rare but may include:

Most serious toxicity is retinopathy, which is associated with long term use (i.e. >5 years of use) and is reversible if in its early stages and the drug is discontinued.

Serious heart rhythm problems may occur, including life-threatening irregular heartbeat. Symptoms can include irregular heartbeats that feel rapid or pounding, chest pain, sudden fainting or feeling tired, light-headed and dizzy, or seizure. The effects on heart rhythm are more likely in high doses of the drug and in combination with other drugs that may cause this problem. All efforts will be made to exclude participants at risk of this problem before entering the trial, and through ongoing monitoring throughout the trial.

Other serious but rare and reversible side effects include changes in your liver enzymes, hypoglycemia and anemia in patients with G6PD deficiency. These resolve once the drug is discontinued.

WHAT ARE THE REPRODUCTIVE RISKS?

The effects that HCQ may have on an unborn baby (fetus) are very rare, however data is very limited. This drug is used routinely to treat patients with Systemic Lupus Erythematosus (SLE) and other inflammatory conditions who are pregnant or breastfeeding. Health Canada has not approved the use of HCQ for the purposes of this study for women who are pregnant, planning to become pregnant or breastfeeding.

Women of childbearing potential will be counselled on contraceptive use. If you become pregnant during the study period, you should notify the study doctor and you will be asked to stop the study drug. The study doctor will ask if you are willing to provide information about the pregnancy as part of this study. You may choose not to give consent for the collection of this information or may withdraw consent at any time without giving a reason. This will not impact your participation on the study and will not result in any penalty or affect your current or future health care.

There are no known reproductive risks for a male partner being on the study drug to either their pregnant partners or if they father a child during the study.

WHAT ARE THE BENEFITS OF PARTICIPATING IN THIS STUDY?

If you agree to take part in this study, the experimental intervention may or may not be of direct benefit to you. You will not benefit from the placebo used in this study. We hope the information learned from this study will help other people exposed to suspected or confirmed COVID-19 in the future.

HOW WILL PARTICIPANT INFORMATION BE KEPT CONFIDENTIAL?

If you decide to participate in this study, the study doctors and study staff will only collect the information they need for this study.

Records identifying you will be kept confidential and, to the extent permitted by the applicable laws, will not be disclosed or made publicly available, except as described in this consent document.

Authorized representatives of the following organizations may look at your original (identifiable) study records at the site where these records are held, to check that the information collected for the study is correct and follows proper laws and guidelines.
The research ethics board who oversees the ethical conduct of this study in Ontario
This institution and affiliated sites, to oversee the conduct of research at this location
Health Canada (because they oversee the use of drugs in Canada)

Information that is collected about you for the study (called study data) may also be sent to the organizations listed above. Representatives of Clinical Trials Ontario, a not-for-profit organization, may see study data that is sent to the research ethics board for this study. Your name, address, email, or other information that may directly identify you will not be used. The records received by these organizations may contain your participant code, sex, partial date of birth.

All positive results for communicable diseases, including COVID-19, must be reported to the Medical Officer of Health (also known as the local public health unit), under the Health Protection and Promotion Act. Additionally, the Ontario government has passed a regulation authorizing first responders, such as police, firefighters and paramedics to access an individual's name, address, date of birth and whether the individual has had a positive test for COVID-19. This regulation may be enlarged or rescinded in the future as the COVID-19 situation changes.

Communication via e-mail is not absolutely secure. We do not recommend that you communicate sensitive personal information via e-mail.

If the results of this study are published, your identity will remain confidential. It is expected that the information collected during this study will be used in analyses and will

be published and presented to the scientific community in meetings and in journals. This information may also be used as part of a submission to regulatory authorities around the world to support the approval of the study intervention.

Even though the likelihood that someone may identify you from the study data is very small, it can never be completely eliminated.

WILL FAMILY DOCTORS/HEALTH CARE PROVIDERS KNOW WHO IS PARTICIPATING IN THIS STUDY?

Your family doctor/health care provider will not be informed by the study team that you are taking part in the study. You can choose to let your family doctor/health care provider know, if you like.

WILL INFORMATION ABOUT THIS STUDY BE AVAILABLE ONLINE?

A description of this clinical trial will be available on https://www.isrctn.com/ and clinicaltrials.gov. These websites will not include information that can identify you. You can search these websites at any time.

WHAT IS THE COST TO PARTICIPANTS?

HCQ and/or placebo will be supplied at no charge while you take part in this study.

It is possible that the supply of HCQ may be limited while you are on the study. Although not likely, if this occurs, your study doctor will speak to you about your options.

You may not be able to receive the study intervention after your participation in the study is completed. There are several possible reasons for this, some of which are:
The intervention may not turn out to be effective or safe.
The intervention may not be approved for use in Canada.
Your health care provider may not feel it is the best option for you.
You may decide it is too expensive and insurance coverage may not be available.
The intervention, even if approved in Canada, may not be available free of charge.

The study doctor will talk to you about your options.

Taking part in this study may result in added costs to you. For example:
There may be extra costs that are not covered by your private insurer /medical plan to treat potential side effects.
There may be costs associated with hospital clinic visits for follow-up. For example, parking or transportation.

ARE STUDY PARTICIPANTS PAID TO BE IN THIS STUDY?

You will not be paid for taking part in this study. In the case of research-related side effects or injury, medical care will be provided by your doctor, or you will be referred for appropriate medical care and follow up.

WHAT ARE THE RIGHTS OF PARTICIPANTS IN A RESEARCH STUDY?

You will be told, in a timely manner, about new information that may be relevant to your willingness to stay in this study.

You have the right to be informed of the results of this study once the entire study is complete. The results of this study will be available on the clinical trial registry.

Your rights to privacy are legally protected by federal and provincial laws that require safeguards to ensure that your privacy is respected.

By signing this form, you do not give up any of your legal rights against the study doctor, sponsor or involved institutions for compensation, nor does this form relieve the study doctor, sponsor or their agents of their legal and professional responsibilities.

You will be given a copy of this signed and dated consent form prior to participating.

WHOM DO PARTICIPANTS CONTACT FOR QUESTIONS?

If you have questions about taking part in this study, or if you suffer a research-related injury, you can talk to your study doctor, or the doctor who is in charge of the study at this institution. That person is:

Name:
Telephone:

Site Study Co-ordinator:
Telephone:

If you have questions about your rights as a participant or about ethical issues related to this study, you can talk to someone who is not involved in the study at all. That person is:

Name: University Health Network Research Ethics Board
Telephone: 416-581-7849

SIGNATURES

Please check all that apply:

All of my questions have been answered,
I understand the information within this informed consent form,
I do not give up any legal rights by signing this consent form,
I allow transfer of specimens and related personal health information as explained in this consent form,
I agree to take part in this study,
I agree to be contacted by telephone or email for the purposes of collecting data,
I agree to follow-up by telephone or email if I miss a study visit,
I agree to my alternate contact being contacted in the event I cannot be reached,

Please check all that apply (optional participation):

I agree to participate in the one month open-label extension of this study,
I agree to the storage and use of my samples for future studies,
I agree to the storage and use of my data for future studies,
I agree to be contacted after the study to be offered participation in future studies.

____________________________________ __________________
Signature of Participant PRINTED NAME Date

_____________________ __________________ __________________
Signature of Person Conducting the Consent Discussion PRINTED NAME & ROLE Date

Please note: More information regarding the assistance provided during the consent process should be noted in the medical record for the participant if applicable, noting the role or relationship of the impartial witness.

---

[i] "WHO Director-General's opening remarking at the media briefing on COVID-10." World Health Organization (WHO) [Press release]. March 11, 2020. [Accessed Mar 21, 2020].

[ii] Bein S. "Coronavirus Update: Provinces threaten fines, arrests to enforce isolation; Vancouver bans most visitors to long-term care homes." The Globe and Mail. Mar 21, 2020. Available at: https://www.theglobeandmail.com/canada/article-coronavirus-update-provinces-threaten-fines-arrests-to-enforce/. [Accessed Mar 22, 2020].

[iii] Ofner-Agostini M, Gravel D, McDonald L. C, Lem M, Sarwal, S, McGreer A, Green K, Vearncombe M, Roth V, Paton S, Loeb M, Simor A, Cluster of Cases of Severe Acute Respiratory Syndrome Among Toronto Healthcare Workers After Implementation of Infection Control Precautions: A Case Series. Hospital Control & Hospital Epidemiology, April 26, 2006.

[iv] Biot C, Daher W, Chavain N, Fandeur T, Khalife J, Dive D, et al. Design and synthesis of hydroxyferroquine derivatives with antimalarial and antiviral activities. *J Med Chem*. 2006;49:2845-2849.
[v] Marmor MF, Kellner U, Lai TY, Melles RB, Mieler WF; American Academy of Ophthalmology. Recommendations on Screening for Chloroquine and Hydroxychloroquine Retinopathy (2016 Revision). *Ophthalmology.* 2016;123(6):1386-1394.
[vi] Yao X, Ye F, Zhang M, Cui C, Huang B, Niu P, et al. In Vitro Antiviral Activity and Projection of Optimized Dosing Design of Hydroxychloroquine for the Treatment of Severe Acute Respiratory Syndrome Coronavirus 2 (SARS-CoV-2). *Clin Infect Dis*. 2020. doi:10.1093/cid/ciaa237. [Epub ahead of print].
